# Does sleep benefit source memory? Investigating 12-h retention intervals with a multinomial modeling approach

**DOI:** 10.3758/s13421-024-01579-8

**Published:** 2024-06-03

**Authors:** Sabrina Berres, Edgar Erdfelder, Beatrice G. Kuhlmann

**Affiliations:** Department of Psychology, School of Social Sciences, University of Mannheim, L13, 15-17, Room 425, 68161 Mannheim, Germany

**Keywords:** Episodic memory, Source memory, Consolidation during sleep, Binding, Multinomial processing tree modeling

## Abstract

For retention intervals of up to 12 h, the active systems consolidation hypothesis predicts that sleep compared to wakefulness strengthens the context binding of memories previously established during encoding. Sleep should thus improve source memory. By comparing retention intervals filled with natural night sleep versus daytime wakefulness, we tested this prediction in two online source-monitoring experiments using intentionally learned pictures as items and incidentally learned screen positions and frame colors as source dimensions. In Experiment 1, we examined source memory by varying the spatial position of pictures on the computer screen. Multinomial modeling analyses revealed a significant sleep benefit in source memory. In Experiment 2, we manipulated both the spatial position and the frame color of pictures orthogonally to investigate source memory for two different source dimensions at the same time, also allowing exploration of bound memory for both source dimensions. The sleep benefit on spatial source memory replicated. In contrast, no source memory sleep benefit was observed for either frame color or bound memory of both source dimensions, probably as a consequence of a floor effect in incidental encoding of color associations. In sum, the results of both experiments show that sleep within a 12-h retention interval improves source memory for spatial positions, supporting the prediction of the active systems consolidation hypothesis. However, additional research is required to clarify the impact of sleep on source memory for other context features and bound memories of multiple source dimensions.

**Supplementary Information:**

The online version contains supplementary material available at 10.3758/s13421-024-01579-8.

## Introduction

Episodic memory refers to memory for past events, experiences, or the source (context)^1^ of information (e.g., location, time; Tulving, 2002). Empirical evidence from neuroimaging techniques such as functional magnetic resonance imaging (fMRI) points to a crucial role of the hippocampus in episodic memory (for reviews, see Eichenbaum et al., 2007; Mitchell & Johnson, 2009). Specifically, the hippocampus appears to bind the content of memories (i.e., item memory) to its unique context (i.e., source memory) during encoding (e.g., Dudai et al., 2015; Mitchell & Johnson, 2009).

Our present research addresses the role of sleep in these source-binding processes. Almost a century of research in neuroscience and psychology has impressively shown that episodic memory is supported by sleep (for a recent meta-analysis, see Berres & Erdfelder, 2021). One mechanism assumed to underlie the sleep benefit in episodic memory is memory consolidation. As such, memory consolidation during sleep increases episodic memory storage by converting recently encoded and therefore labile memories into more stable long-term memory representations (Buzsáki, 1998; Diekelmann & Born, 2010; Dudai, 2004, 2012; Dudai et al., 2015; Klinzing et al., 2019; Rasch & Born, 2013). There are various theories that explain sleep benefits in episodic memory by memory consolidation, such as the sequential hypothesis^2^ (Ambrosini & Giuditta, 2001; Giuditta, 2014; Giuditta et al., 1995) and the synaptic homeostasis hypothesis^3^ (Cirelli & Tononi, 2015; Tononi & Cirelli, 2003, 2006, 2014, 2020). In the current work, we focus on memory consolidation as proposed by the active systems consolidation hypothesis (Born & Wilhelm, 2012; Diekelmann & Born, 2010; Feld & Born, 2017; Inostroza & Born, 2013; Klinzing et al., 2019; Rasch & Born, 2013). This hypothesis is arguably “the currently most integrative account of sleep-dependent memory consolidation” (Klinzing et al., 2019, p. 1598), because it incorporates aspects of various consolidation theories – including the sequential and synaptic homeostasis hypothesis. Specifically, the active systems consolidation hypothesis states that during wakefulness, components of a memory representation (e.g., color, texture, odor of a fruit) are formed and distributed across various neocortical brain areas. In parallel, the hippocampus binds these components to a unique memory representation (e.g., Feld & Born, 2017; Klinzing et al., 2019). During subsequent sleep, especially during slow-wave sleep (SWS), the hippocampal memory representation is replayed by reactivating specific neuronal firing patterns (Klinzing et al., 2019; Lewis & Durrant, 2011; O’Neill et al., 2010; Pfeiffer, 2020; Wilson & McNaughton, 1994). These local synaptic upscaling processes strengthen not only synaptic connections in the hippocampus, and thus stabilize the hippocampal memory representation, but also strengthen the separate components of the memory representation by triggering replay in various neocortical brain areas. Simultaneously, global synaptic downscaling renormalizes the strength of synaptic connections across all cortical and subcortical areas by diminishing neuronal firing rates (Feld & Born, 2017; Klinzing et al., 2019). It is assumed that the combination of local synaptic upscaling and global synaptic downscaling in the hippocampus and neocortex results in a net strengthening of episodic context-bound hippocampal memory representations for relatively short retention intervals (e.g., 12 h) and more gist-like decontextualized neocortical memory representations for longer retention intervals (e.g., 3 days; Klinzing et al., 2019). This assumption is supported by studies indicating a strengthening but no decontextualization of episodic memories within 10–12 h after learning (e.g., Jurewicz et al., 2016; Lutz et al., 2017). In brief, according to the active systems consolidation hypothesis, sleep compared to wakefulness within a 12-h retention interval should strengthen associations between the components of a memory representation that were previously established during encoding.

To investigate the sleep benefit in episodic memory, researchers have often used item-item associations such as word pairs as stimulus material (Diekelmann et al., 2009; Klinzing et al., 2019; for a meta-analysis on single words and word pairs, see Berres & Erdfelder, 2021). By contrast, only a few studies investigated the sleep benefit using item-source associations (for a discussion of functional differences between item-item and item-source associations, see Mayes et al., 2007). In the following section, we review the rather mixed outcomes of studies addressing sleep benefits in memory for item-source associations.

## Overview of research on sleep benefits in source memory

Using a split-night design, Rauchs et al. (2004) found better free recall performance for spatial positions (i.e., top vs. bottom) of words in a what-where-when task after sleep in the second half of the night (predominantly characterized by rapid eye movement (REM) sleep) compared to wakefulness. In contrast, sleep–wake comparisons in the second half of the night for word-list associations (i.e., temporal source memory, “when” dimension) showed no significant differences. Correspondingly, the authors found no significant differences for sleep–wake comparisons in the first half of the night (predominantly characterized by SWS) for spatial positions and lists. Furthermore, all sleep–wake comparisons for spatial positions and lists in the subsequent recognition test were not significant. When comparing sleep deprivation in the first versus the second half of the night, the authors found better free recall performance for word positions after SWS deprivation than after REM sleep deprivation (Rauchs et al., 2004). These results suggest that REM sleep contributes to the sleep benefit in item-position associations, thereby conflicting with the active systems consolidation hypothesis, which considers SWS to be more important for memory consolidation. However, in line with the consolidation hypothesis, other split-night studies showed worse memory of the frame color and spatial position for neutral pictures after REM-rich late sleep than after SWS-rich early sleep, pointing to a pivotal role of SWS for memory performance (see Groch et al., 2015; Sopp et al., 2017).

The results were also mixed for studies comparing naps versus wakefulness during the day or early evening: Wang and Fu (2009) as well as Köster et al. (2017) found no significant differences between naps and wakefulness for picture-background color associations, contradicting the active systems consolidation hypothesis. By contrast, van der Helm et al. (2011) found a significant sleep benefit in source memory for word-context associations after naps in line with the active systems consolidation hypothesis. Further support is provided by Lewis et al. (2011, Experiment 2), who observed significantly less forgetting after naps compared to wakefulness in source memory for object-background photo associations.

Classical sleep study designs that compared night-time sleep and daytime wakefulness using retention intervals up to 12 h resulted in somewhat stronger evidence for sleep-induced context memory improvements as predicted by the active systems consolidation hypothesis. Lewis et al. (2011) made use of such a design in their first experiment and found significantly less forgetting of encoding contexts after night-time sleep than daytime wakefulness, very similar to their nap study results in Experiment 2. Also using a retention interval of 12 h filled with either sleep or wakefulness, Mawdsley et al. (2014) observed a significant sleep benefit in source memory for word-position associations. Wang et al. (2017) investigated the sleep benefit for word pair-temporal context associations in children. Specifically, children learned two lists of word pairs separated by a 1-h delay between learning of the first and second list (temporal context). After a retention interval of 11 h, memory for word pairs was tested with a cued recall task. In addition, children were asked to indicate the list of the respective word pair. Interpolated sleep compared to wakefulness improved memory for word pairs and the temporal context but not for word pair-temporal context associations (Wang et al., 2017). Hence, this result provides no support for the prediction of the active systems consolidation hypothesis that sleep compared to wakefulness within a 12-h retention interval improves source memory for word-pair-context associations.

Overall, the empirical evidence concerning sleep benefits in source memory is thus quite mixed. The reviewed studies differ in several aspects that may explain the mixed results observed with respect to the sleep benefit in item-context associations. For example, researchers have not only used a wide variety of sleep study designs (i.e., split-night designs, daytime naps, night-time naps, natural sleep and wakefulness), but also different item materials (i.e., single words, word pairs, pictures) and sources (i.e., spatial positions, frame colors, background colors, background photos, posters, lists), next to different encoding instructions (i.e., intentional learning of item-context associations, incidental learning of item-context associations, intentional learning of items but incidental learning of contexts), participant populations, sample sizes, and experimental designs (i.e., within-subjects design, between-subjects design; see Appendix Table 5 for an overview of study characteristics).

Furthermore, the variety of source memory measures used likely contributed to the inconsistent results. According to the source-monitoring framework, multiple cognitive processes such as memory, decision making, guessing, and response biases are involved in making judgments about the origin of a memory (Johnson et al., 1993). These cognitive processes are confounded in frequently used standard measures of source memory (cf. Batchelder & Riefer, 1990). Source memory is often measured by simply counting the number of correct source attributions (e.g., Groch et al., 2015; Lewis et al., 2011; Mawdsley et al., 2014; Wang et al., 2017) or by using the source-identification measure (SIM; e.g., van der Helm et al., 2011), defined as the proportion of correct source attributions for all target items, irrespective of whether they were identified as “old” or “new.” Another frequently used measure for source memory is the average conditional source identification measure (ACSIM; Rauchs et al., 2004; Sopp et al., 2017; Wang & Fu, 2009), defined as the proportion of correct source attributions for all target items correctly identified as “old,” averaged across the two sources (e.g., left, right). Although item and source memory are somewhat less confounded in ACSIM than in SIM, all these source memory measures confound item memory, source memory, and guessing to some degree (Bröder & Meiser, 2007; Murnane & Bayen, 1996). We therefore argue that more rigorous and less contaminated measures of source memory are required to test whether sleep compared to wakefulness strengthens the context binding of episodic memories for retention intervals up to 12 h, as predicted by the active systems consolidation hypothesis (Inostroza & Born, 2013; Klinzing et al., 2019). Multinomial processing tree (MPT) models of source monitoring (Batchelder & Riefer, 1990; Bayen et al., 1996; Meiser & Bröder, 2002) provide an appropriate framework to achieve this goal. However, to our knowledge, sleep benefits in source memory have not yet been investigated using such models so far. In the current work, we aim to fill this gap by testing the sleep-strengthens-source-memory hypothesis using validated MPT measures of source memory tailored to two different source-monitoring tasks. Of course, to ensure comparability with previous research, traditional measures of item and source memory are employed in addition.

## The current experiments

In Experiment 1, we manipulated the spatial position of pictures on a computer screen in a standard source-monitoring task (e.g., Bayen et al., 1996; Murnane & Bayen, 1996) to investigate source memory for item-context associations after a 12-h retention interval filled with either a period of night-time sleep or daytime wakefulness. We conducted a second experiment with the main purpose of conceptually replicating the results for spatial position memory of Experiment 1. In Experiment 2, we additionally manipulated the frame color orthogonally to the spatial position of pictures. This allowed us to explore two additional research questions: First, does the result for spatial position memory generalize to other source dimensions (i.e., frame color memory)? Second, does sleep compared to wakefulness benefit memory for context-context associations (i.e., bound source memory for spatial position and frame color)?

In both experiments, we explicitly instructed participants to study pictures for a later recognition test (i.e., intentional learning of items), whereas no such instruction was provided for their sources (i.e., sources were learned incidentally). To counteract possible floor effects in source memory, participants performed an orienting task during the learning phase that requires attending to the relevant source information but involves no rehearsal (i.e., indicating spatial positions using response keys during stimulus presentation on the screen; cf. Boywitt & Meiser, 2012). By preventing participants from using explicit rehearsal strategies for item-context and context-context associations, this approach creates a more realistic setting for examining everyday source monitoring. Note that most previous studies on the sleep benefit in context-binding employed intentional learning of item-context associations (for incidental learning, e.g., see Mawdsley et al., 2014; Wang et al., 2017).

To allow comparisons with previous studies, we report hit rates and false-alarm rates in addition to the sensitivity index *d’* and response bias *c* for item memory. Whereas sensitivity and response bias are confounded in hit rates (i.e., proportion of target items correctly identified as “old”) and false-alarm rates (i.e., proportion of distractor items falsely identified as “old”), sensitivity and response bias are separated in *d’* and *c* as derived from the signal detection theory (SDT; Stanislaw & Todorov, 1999; e.g., van der Helm et al., 2011). Specifically, larger positive values of *d’* indicate better discrimination between target and distractor items. Response bias* c* denotes the general response tendency, with larger negative values indicating a stronger “old”-response bias, values close to zero no response bias, and larger positive values a stronger “new”-response bias (Stanislaw & Todorov, 1999).

For source memory, we report the average conditional source identification measure (ACSIM), defined as the proportion of correct source attributions for all target items correctly identified as “old,” averaged across the two sources (e.g., left, right) of a source dimension (e.g., spatial position; Murnane & Bayen, 1996). Because ACSIM is not defined when all target items correctly identified as “old” are assigned to the same source (e.g., right) of a source dimension (e.g., spatial position), we report the conditional source identification measure (CSIM) in these cases. This measure is defined as the averaged proportion of correct source attributions for all target items correctly identified as “old.” For ACSIM and CSIM, larger positive values indicate better source memory. Note, however, that both measures confound source memory with item memory in some circumstances, for example, when targets are identified as “old” based on guessing (Bayen et al., 1996; for a detailed discussion, see Murnane & Bayen, 1996).

In contrast to ACSIM and CSIM, MPT models allow us to disentangle source memory from item memory and guessing (for reviews on this model class and a MPT tutorial, see Batchelder & Riefer, 1999; Erdfelder et al., 2009; Schmidt et al., 2023). MPT models have therefore gained considerable popularity in source memory research in general (e.g., Arnold et al., 2019; Bell et al., 2012, 2017; Boywitt & Meiser, 2012; Kuhlmann et al., 2016; Nadarevic & Erdfelder, 2013, 2019), albeit with the exception of sleep-related research.

There are several options for fitting MPT models to empirical data (e.g., Heck et al., 2018; Moshagen, 2010; Nestler & Erdfelder, 2023), with complete and partial pooling being the two most often used methods. Specifically, in the complete pooling approach, observed category frequencies are aggregated across participants, and the maximum likelihood (ML) method is used to obtain MPT-parameter estimates (aggregated model-based analysis). In contrast to complete pooling, the partial pooling approach explicitly accounts for individual differences between participants by combining information on the individual and group level (hierarchical model-based analysis). For individual and group-level parameter estimation, partial pooling often relies on a Bayesian approach employing Markov-chain Monte Carlo (MCMC) methods (Heck et al., 2018). Here we performed both aggregated and Bayesian hierarchical model-based analyses to check whether our results are robust against the different distributional assumptions involved in complete and partial pooling. For complete pooling in the aggregated model-based analysis, we used the software multiTree (Moshagen, 2010). The latent-trait approach (Klauer, 2010) as implemented in the R package TreeBUGS (Heck et al., 2018) was used for partial pooling in the hierarchical model-based analysis.

Hypotheses, study design, sample size, and analysis plan were preregistered for Experiment 1 (https://osf.io/gctzn) and Experiment 2 (https://osf.io/a6z4u). For both experiments, the data and stimulus materials are available via the Open Science Framework (OSF; https://osf.io/8rmj2/?view_only=02e5eec5c3e54fd4aff3d55eedebffa7). In the respective *Method* sections, we provide detailed information about the MPT models used, sample size determination, and data exclusions.

## Experiment 1

**Fig. 1:**
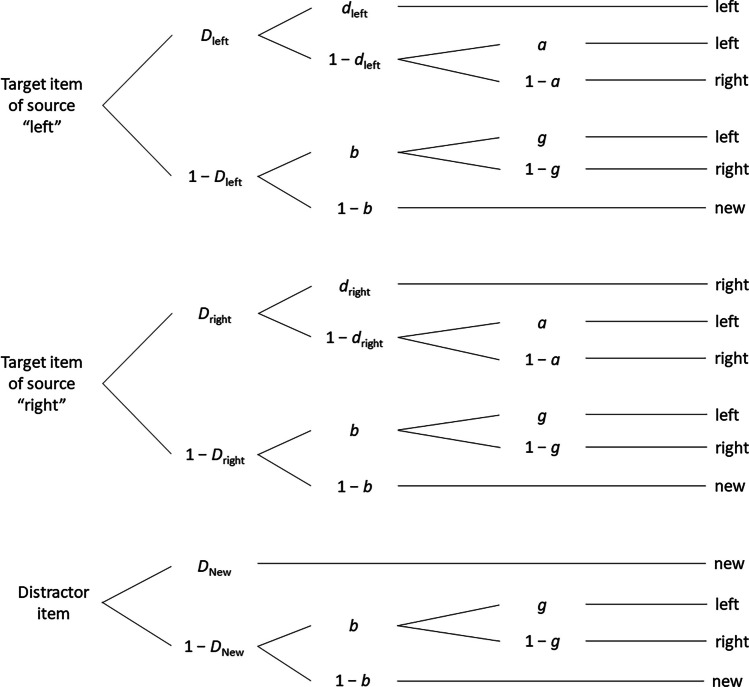
Two-high-threshold multinomial model of source monitoring (2HTSM) adapted to the spatial position source manipulation used in Experiment 1. *D*_left_ = probability of correctly identifying a target item in source “left” as “old”; *D*_right_ = probability of correctly identifying a target item in source “right” as “old”; *D*_New_ = probability of correctly identifying a distractor item as “new”; *d*_left_ = probability of correctly identifying the source of a target item in source “left”; *d*_right_ = probability of correctly identifying the source of a target item in source “right”; *a* = probability of guessing that a correctly identified target item is from source “left”; *b* = probability of guessing that an item is “old”; *g* = probability of guessing that an unrecognized item is from source “left” if it was guessed to be “old”. Adapted from “Source Discrimination, Item Detection, and Multinomial Models of Source Monitoring,” by U. J. Bayen, K. Murnane, and E. Erdfelder, 1996, *Journal of Experimental Psychology: Learning, Memory, and Cognition, 22*(1), p. 202 (https://doi.org/10.1037/0278-7393.22.1.197). Copyright 1996 by the American Psychological Association

To reiterate, according to the active systems consolidation hypothesis, the hippocampus binds the content (i.e., item memory) and its unique context (i.e., source memory) to a unique memory representation during encoding. This memory representation is replayed during subsequent sleep, which should result in better item and source memory compared to wakefulness (Feld & Born, 2017; Klinzing et al., 2019). For a 12-h retention interval, the active systems consolidation hypothesis thus predicts that both item memory and source memory should benefit from sleep. To test these two hypotheses, we used the two-high-threshold MPT model of source monitoring (2HTSM) shown in Fig. 1. The 2HTSM model performed best in a comparative validation study of source-monitoring models (Bayen et al., 1996). As such, this model is based on a standard source-monitoring task in which participants study items from two sources and are subsequently asked whether the item was previously presented, and if so, in which source (e.g., Bayen et al., 1996; Murnane & Bayen, 1996). The 2HTSM provides separate parameters for item memory, source memory, and guessing. Specifically, participants correctly recognize a target item presented in source A or B as “old” or a distractor item as “new” with probability *D*. Conditionally on correct item recognition, participants correctly identify the source with probability *d*. However, if item memory (1—*D*) or source memory (1—*d*) fails, participants are assumed to guess. In case of successful item memory but failing source memory, participants correctly guess the source of a target item with probability *a*. If item memory fails, participants guess “old” with probability *b*. Finally, if both item and source memory fail, participants correctly guess the source with probability *g* (Bayen et al., 1996).

In the most general version of the 2HSTM, item memory, source memory, and source guessing may vary between item types and sources as illustrated in Fig. 1. To arrive at an identifiable and most parsimonious 2HTSM submodel that still fits the data, we first tested invariance of item memory with respect to item types and sources, followed by invariance tests of source memory, and finally guessing. By using this principled strategy, we aimed at identifying a submodel with a minimum of precisely estimable parameters (see Bayen et al., 1996).

According to the active systems consolidation hypothesis, the corresponding item memory (*D*) and source memory (*d*) parameters should both be larger when participants sleep during the 12-h retention interval than when they stay awake.

### Method

In this experiment, we compared participants randomly assigned to a wake versus sleep condition. Whereas participants in the wake condition learned the material in the morning and were tested in the evening after a 12-h retention interval of daytime wakefulness, this was reversed for participants in the sleep condition, who were tested after a period of night-time sleep. Crucially, note that previous research showed comparable performance in learning as well as testing parameters by using the same sleep study design, showing that circadian effects are not a serious confound in this design (e.g., Abel & Bäuml, 2012, 2013a, 2013b, 2014; Bäuml et al., 2014; Erdfelder et al., 2024; Fenn & Hambrick, 2013).

#### Participants

We determined the necessary sample size a priori by conducting two power analyses: First, despite our directional predictions, we conservatively performed an a priori power analysis for a two-tailed *t* test with two independent groups using G*Power 3.1 (Faul et al., 2007). Given a medium effect size (Cohen’s **d** = 0.50), a conventional α-level of 0.05, and a target-power of 1—β = 0.80, the analysis resulted in a total sample size of 128 participants. Second, we determined the necessary sample size for the model-based analysis using multiTree (Moshagen, 2010). Assuming a sleep–wake difference of 0.10 in the crucial parameter (*D* or *d*, depending on the hypothesis), an analysis based on 130 participants, 60 target items, and 30 distractor items resulted in a power larger than 0.99 for the item memory parameter *D* and a power of 0.96 for the source memory parameter *d* (for more detailed information, see the preregistration on the OSF, https://osf.io/gctzn). Thus, we strove for a sample of 130 participants. Data collection took place from fall 2020 to spring 2021. Note that we extended the data collection phase until we reached the desired number of participants because data collection was slow and only a fraction of the targeted sample size was collected within the preregistered 3 months.

In total, 174 participants recruited via mailing lists of the University of Mannheim, social media, personal contacts, and the online research platform Prolific (https://www.prolific.co; Palan & Schitter, 2018; Peer et al., 2017) took part in the online experiment. To participate in the experiment, participants had to be between 18 and 35 years old, speak German fluently, and have no neurological disorders (see the preregistration on the OSF, https://osf.io/gctzn). After successful completion of the experiment, 103 participants recruited via Prolific (59.20%) were paid a flat fee of £4.50, whereas 71 participants recruited through other channels (40.80%) either received corresponding course credits or were eligible to win vouchers. Due to random assignment to the wake versus sleep condition, the number of participants who were paid (*n*_wake_ = 50, *n*_sleep_ = 53), received corresponding course credits, or were eligible to win vouchers (*n*_wake_ = 40, *n*_sleep_ = 31) were approximately balanced across the experimental conditions.^4^ Note that the experiment was successfully completed only if the following two conditions were met: First, all parts of the experiment had to be completed within the set time frames (i.e., registration, learning, and testing session). Second, more than 50% of the responses in the orienting task had to be correct.

Following the preregistered exclusion criteria, 23 participants were excluded from the analysis, because they indicated that they were distracted or interrupted during the experiment. Another four participants had to be excluded because the retention interval was not within 11–13 h. Furthermore, seven participants of the wake condition were excluded because they napped during the retention interval, and two participants were excluded because they reported having neurological disorders. We also excluded two participants because of substantial alcohol consumption during the retention interval (i.e., females were excluded if they consumed more than 20 g alcohol, males were excluded if they consumed more than 40 g alcohol), and one participant with a larger false-alarm rate than hit rate. Three additional participants were excluded for unforeseen reasons not included in the preregistration: One participant reported using memory aids (e.g., notes, screenshots), one participant reported technical problems, and another participant assigned to the wake condition delayed the start of the experiment so that it started in the evening instead of the morning. In sum, we excluded 42 participants, leaving 132 participants (*n*_wake_ = 65, *n*_sleep_ = 67) for analysis, all of them fluent in German. The 132 participants were between 18 and 35 years of age (*M* = 26.77 years, *SD* = 4.48), 84 (63.64%) were female. For all participants, many more than the minimally required 50% of the responses in the orienting task were correct (*M*_total_ = 98%, *M*_wake_ = 97%, *M*_sleep_ = 98%; see Table S1 in the Online Supplemental Materials (OSM) for more detailed sample characteristics), confirming that they paid attention to the source (i.e., spatial position) at encoding.

#### Materials

We selected 160 colored object photos from the bank of standardized stimuli (BOSS; Brodeur et al., 2010) of which 60 randomly chosen target pictures were displayed on either the left or the right side of the screen (i.e., 30 pictures each were displayed at the 10% and 90% position on the x-axis). Thus, spatial positions of pictures (left vs. right) served as the two sources of interest. Another 30 pictures were randomly selected as distractors, and four additional pictures were randomly selected as buffer items that were included at the start of the learning phase to prevent primacy effects. Note that we decided against including a recency buffer because of the 12-h retention interval. A list of the 160 pictures and detailed information about the selection criteria are available via the OSF (https://osf.io/8rmj2/?view_only=02e5eec5c3e54fd4aff3d55eedebffa7).

#### Procedure

**Fig. 2:**
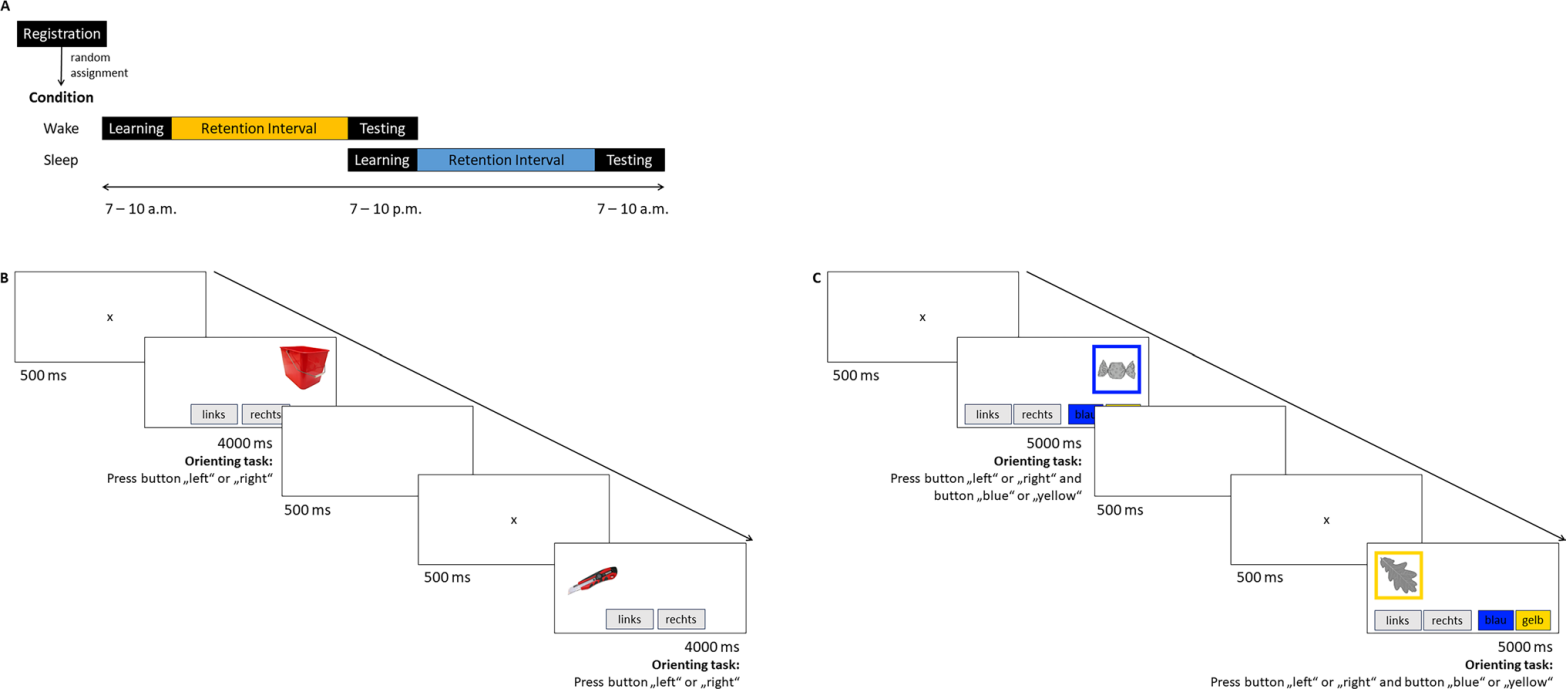
Procedure of the online experiments in Experiment 1 and Experiment 2. **Panel A:** The learning session in Experiment 1 contained 64 colored object photos (i.e., four buffer and 60 target items) presented left versus right whereas the learning session in Experiment 2 contained 124 grey-scaled object drawings (i.e., four buffer and 120 target items) presented left versus right and in a blue versus yellow frame. For each item in the testing session of Experiment 1, participants made an “old”–”new” decision, followed by a “left” – “right” decision for an “old”-response. For each item in the testing session of Experiment 2, participants made an “old” – “new” decision, followed by a “left plus blue” – “left plus yellow” – “right plus blue”- “right plus yellow” decision for an “old”-response. In both experiments, this was followed by control- and demographic questions. **Panel B:** Orienting task during the study phase in Experiment 1. **Panel C:** Orienting task during the study phase in Experiment 2. See the online article for the color version of this figure

The online experiment was conducted with SoSci Survey (Leiner, 2020), using lab.js (Henninger et al., 2022) for stimulus presentation during the study phase, and consisted of three parts: registration, learning, and testing session (see Fig. 2 for an illustration). In the registration session, participants gave informed consent before being randomly assigned to either the sleep or wake condition. They were asked to pick a date and time for the first session in line with their randomly predetermined condition (i.e., wake condition: 7 a.m. to 10 a.m.; sleep condition: 7 p.m. to 10 p.m.) and were informed that the second session starts 12 h later. Participants received the access link via email or Prolific notification 15 min before the start of the learning session. During the study phase, 64 randomly selected pictures (i.e., four buffer and 60 target items) were sequentially presented on the left or right side of the screen for 4 s each with an interstimulus interval of 1 s (i.e., blank white screen for 500 ms followed by a fixation cross for 500 ms). While a picture was presented on the screen, participants performed the orienting task, which entailed pressing the correct button for the spatial position within the 4-s picture-presentation time. The two buttons labeled “left” and “right” were arranged next to each other and were displayed below the picture. Only participants who answered with the correct spatial position for more than 50% of the 64 pictures completed the learning session and were invited to the testing session 12 h later. Again, participants received the access link via email or Prolific notification 15 min before the session started. For the testing session, the 60 target items were intermixed with 30 distractor items and presented in the middle of the screen with two buttons labeled “old” and “new” below. Note that we varied the spatial position of the labels “old” and “new” randomly between participants but kept it constant within participants. By pressing one of the two buttons, participants indicated whether the picture was presented during the study phase (“old”) or not (“new”). If participants answered “old,” they were asked whether the picture was presented left or right and to respond with the corresponding button. This task was followed by control and demographic questions, which also included the exclusion criteria mentioned before (e.g., distraction, alcohol consumption, use of memory aids, technical problems; for details, see the preregistration on the OSF, https://osf.io/gctzn). Finally, participants were thanked and debriefed.

## Results

We set a significance level of α = 0.05 for all analyses. For hit and false alarm rates as well as *d’* and *c* measures of item recognition we report means, standard errors, and *t*-test results in Table 1.^5^ Regarding item memory, all two-tailed *t* tests for two independent groups showed no statistically significant differences between the sleep and the wake condition, *t*(130) ≤ 1.62,* p* ≥ 0.107 (see Table 1). In contrast, source memory as measured by ACSIM significantly benefitted from sleep, *t*(130) = 3.46, *p* = 0.001, estimated Cohen’s **d** = 0.59 (sleep condition: *M* = 0.77, *SE* = 0.01; wake condition: *M* = 0.69, *SE* = 0.01; Table 1).^6^ Taken together, using commonly applied measures of item and source memory, we found statistically significant evidence for a sleep benefit in source memory but not in item memory.

**Table 1:** Results of item- and source-memory analyses in Experiment 1

Dependent variable	Wake		Sleep				
	*M*	*SE*	*M*	*SE*	*t*(130)	*p*	Cohen’s** d** [95% CI]
Item memory							
Hit rate	0.70	0.02	0.72	0.02	0.65	0.517	0.11 [-0.23, 0.45]
False-alarm rate	0.10	0.01	0.07	0.01	1.62	0.107	-0.30 [-0.64, 0.05]
Sensitivity index *d’*	2.05	0.09	2.27	0.08	1.35	0.181	0.24 [-0.11, 0.58]
Response bias *c*	0.41	0.03	0.43	0.03	0.31	0.759	0.05 [-0.29, 0.39]
Source memory							
ACSIM	0.69	0.01	0.77	0.01	3.46	0.001	0.59 [0.24, 0.94]

*Note: *Means and standard errors of the mean are shown for the wake (*n* = 65) and sleep condition (*n* = 67), as well as the results of two-tailed *t* tests comparing the two independent groups. We estimated Cohen’s **d** on the basis of the means and pooled standard deviations. Note that for both item and source memory, positive values of Cohen’s **d** indicate a sleep benefit, whereas negative values indicate a sleep disadvantage compared to wakefulness. ACSIM = average conditional source identification measure

The most parsimonious model we originally aimed at – Submodel 4 of the 2HTSM with parameter *D* for item memory, parameter *d* for source memory, and parameters *b* and *g* for guessing (Bayen et al., 1996; see the preregistration on the OSF, https://osf.io/gctzn) – produced considerable misfit for the aggregated data, *G*^2^(4) = 10.21, *p* = 0.037. While invariance of item and source memory parameters across item types and sources turned out to be unproblematic, assuming invariance of source guessing parameters *a* and *g* in addition resulted in the observed misfit. Hence, applying Submodel 5a of the 2HTSM (Bayen et al., 1996) – with a single parameter *D* for item memory, a single parameter *d* for source memory, and three parameters *a*, *b*, and *g* for guessing – resulted in a good fit, *G*^2^(2) = 1.78, *p* = 0.411. The ML parameter estimates, standard errors, and 95% confidence intervals of Submodel 5a for the wake and sleep condition are summarized in Table 2. We found a statistically significant difference between sleep versus wake conditions in the item memory parameter *D*, Δ*G*^2^(1) = 13.66, *p* < 0.001. The item memory parameter estimate for the sleep condition was almost 5% larger than for the wake condition. Similarly, the source memory parameter *d* also differed significantly between conditions, Δ*G*^2^(1) = 31.30, *p* < 0.001, with about 15% higher source memory estimates after sleep than after wakefulness. Concerning the guessing parameters, we found significantly more “old”-guessing in the wake than the sleep condition (parameter *b*), Δ*G*^2^(1) = 6.09, *p* = 0.014; and a significantly stronger “left” guessing bias for unrecognized items after sleep than after wakefulness (parameter *g*), Δ*G*^2^(1) = 10.13, *p* = 0.001. By contrast, there was no statistically significant difference between the sleep and wake condition in source guessing for recognized items (parameter *a*), Δ*G*^2^(1) = 1.36, *p* = 0.243.

**Table 2:** Aggregated parameter estimates of the two-high-threshold multinomial model of source monitoring (2HTSM) for Experiment 1

Parameter	Wake			Sleep		
	MLE	*SE*	95% CI	MLE	*SE*	95% CI
*D*	0.60	0.01	[0.58, 0.62]	0.65	0.01	[0.64, 0.67]
*d*	0.48	0.02	[0.44, 0.52]	0.63	0.02	[0.60, 0.67]
*a*	0.44	0.02	[0.39, 0.48]	0.48	0.03	[0.43, 0.53]
*b*	0.25	0.01	[0.22, 0.27]	0.20	0.01	[0.17, 0.23]
*g*	0.47	0.04	[0.40, 0.54]	0.64	0.04	[0.57, 0.72]

*Note: *For the aggregated model-based analysis, maximum likelihood estimates (MLE), standard errors (*SE*), and 95% confidence intervals (CI) are reported. D = probability of correctly identifying a target item as “old” and a distractor item as “new”; *d *= probability of correctly identifying the source of a target item; *a* = probability of guessing that a correctly identified target item is from source “left”; *b* = probability of guessing that an item is “old”; *g* = probability of guessing that an unrecognized item is from source “left” if it was guessed to be “old”

To check the robustness of our results, we reanalyzed the same data by performing a hierarchical model-based analysis in the framework of Klauer’s (2010) latent-trait model as implemented in TreeBUGS (Heck et al., 2018) for partial pooling. As can be seen in the Appendix (see Table 6), the estimated group-level means resembled those reported in Table 2. We thus conclude that the basic result pattern does not depend on whether complete or partial pooling approaches are used for data analysis.

## Discussion

Both the ACSIM-based and the model-based results suggest that sleep compared to wakefulness benefits source memory. This is in line with a core prediction of the active systems consolidation hypothesis that sleep benefits source memory for retention intervals of up to 12 h.

For item memory, the descriptive result patterns of *d’* and the aggregated as well as hierarchical model-based analyses suggest that sleep compared to wakefulness might benefit item recognition. Whereas item memory was descriptively higher after sleep versus wakefulness in all three analyses, the sleep benefit was significant only for complete pooling. This deviance is likely due to different analysis-levels (i.e., complete pooling, partial pooling, no pooling) that account for potential individual differences to a varying extend. Specifically, the complete pooling approach (aggregated analysis) assumes that the data are independently and identically distributed for all participants, thereby ignoring potential individual differences. By contrast, the partial pooling approach (hierarchical analysis) accounts for individual differences. The same applies to *d’*, which is calculated for each participant separately (i.e., no pooling). Thus, the significant sleep benefit in item memory observed for complete pooling is likely due to the fact that partial and no pooling approaches account for individual differences, whereas the complete pooling approach does not. Importantly, our mixed results concerning item memory are in line with previous research that uses recognition tasks to assess item memory, also yielding mixed evidence for the active systems consolidation hypothesis: Some studies found a significant sleep benefit in item memory (e.g., Köster et al., 2017; Mawdsley et al., 2014), whereas others did not (e.g., van der Helm et al., 2011; Wang & Fu, 2009). In fact, a recent meta-analysis showed that the sleep benefit for word materials is largest in free recall (Hedges’ *g* = 0.49), followed by cued recall (Hedges’ *g* = 0.45), and lastly recognition tasks (Hedges’ *g* = 0.38; Berres & Erdfelder, 2021). This suggests that item recognition apparently benefits from sleep only slightly, thereby making it difficult to detect these small positive sleep effects in item recognition tasks (e.g., Rauchs et al., 2004; Wang & Fu, 2009).

In sum, Experiment 1 indicates that sleep improves source memory within a 12-h retention interval as predicted by the active systems consolidation hypothesis. However, to establish the validity of this conclusion more rigorously, our results require an experimental follow-up evaluation. We therefore conducted a second experiment with the aim of conceptually replicating the results for spatial position memory. In addition, by manipulating frame color orthogonally to the spatial position of pictures in Experiment 2, we were able to explore whether the results for spatial position memory generalize to a second source dimension (i.e., frame color). Furthermore, we explored whether sleep within a 12-h retention interval also strengthens bound memory for spatial position and frame color.

## Experiment 2

**Fig. 3:**
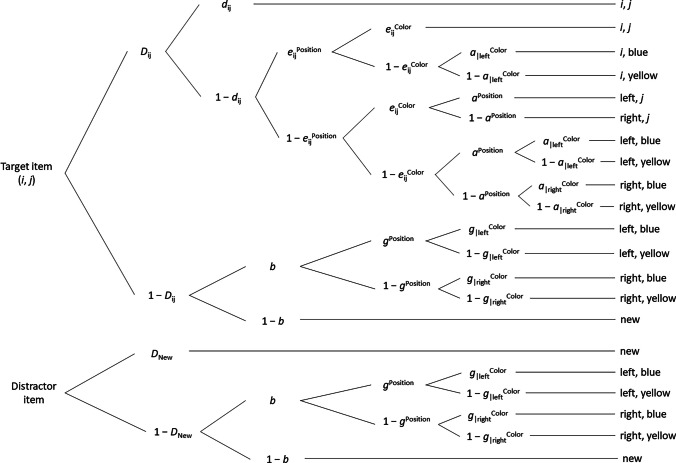
Multinomial model of multidimensional source monitoring used in Experiment 2. *D*_ij_ = probability of correctly recognizing a target item in sources *i* (i.e., “left” or “right” on source dimension “spatial position”) and *j* (i.e., “blue” or “yellow” on source dimension “frame color”) of both source dimensions; *D*_New_ = probability of correctly identifying a distractor item as “new”; *d*_ij_ = probability of correctly identifying the source combination *i*, *j* of a recognized item (i.e., “left and blue,” “left and yellow,” “right and blue,” or “right and yellow,” respectively); *e*_ij_^Position^ = probability of correctly identifying the source (i.e., left, right) on source dimension “spatial position” of a recognized item; *e*_ij_^Color^ = probability of correctly identifying the source (i.e., blue, yellow) on source dimension “frame color” of a recognized item; *a*^Position^ = probability of guessing ‘‘left” on source dimension “spatial position” of a recognized item; *a*_|left_^Color^ = probability of guessing “blue” on source dimension “frame color” if the target item was correctly identified as “old” and assigned to source “left”; *a*_|right_^Color^ = probability of guessing “blue” on source dimension “frame color” if the target item was correctly identified as “old” and assigned to source “right”; *b* = probability of guessing that an unrecognized item is “old”; *g*^Position^ = probability of guessing “left” on source dimension “spatial position” if the unrecognized item was guessed to be “old”; *g*_|left_^Color^ = probability of guessing “blue” on source dimension “frame color” if the unrecognized item was guessed to be “old” and assigned to source “left”; *g*_|right_^Color^ = probability of guessing “blue” on source dimension “frame color” if the unrecognized item was guessed to be “old” and assigned to source “right”. Adapted from “Analyzing Stochastic Dependence of Cognitive Processes in Multidimensional Source Recognition,” by T. Meiser, 2014, *Experimental Psychology, 61*(5), p. 408 (https://doi.org/10.1027/1618-3169/a000261). Copyright 2014 by Hogrefe Publishing

As in Experiment 1, we predict that both item memory and source memory should benefit from sleep compared to wakefulness in a 12-h retention interval. Because hippocampal memory representations include not only item-context but also context-context associations, we also explored whether sleep improves bound memory for two source dimensions. We tested these predictions using a reparameterized variant of the MPT model of multidimensional source monitoring (Meiser, 2014), shown in Fig. 3. Like the 2HTSM, this model is based on a source-monitoring task that is, however, extended to two source dimensions (e.g., a position dimension with sources “left” and “right,” and a color dimension with sources “blue” and “yellow”; Meiser, 2014).

The multinomial model of multidimensional source monitoring provides separate parameter estimates for item memory, bound source memory (i.e., spatial position plus frame color), unbound source memory (e.g., spatial position only), and guessing. Specifically, participants correctly recognize a target item presented by source *i* of the first source dimension (e.g., “left” or “right” on source dimension “spatial position”) and source *j* of the second source dimension (e.g., “blue” or “yellow” on source dimension “frame color”) as “old” with probability *D*_ij_ or detect a distractor item as “new” with probability *D*_new_. Conditionally on correct item recognition, participants correctly identify the source combination (e.g., left and blue, left and yellow, right and blue, right and yellow) of recognized items with bound source probability *d*_ij_. In contrast, if bound source memory fails for recognized items (i.e., the source combination is not correctly identified with probability 1—*d*_ij_), participants can still correctly identify the sources *i* (e.g., “left” or “right” on source dimension “spatial position”) and *j* (e.g., “blue” or “yellow” on source dimension “frame color”) of either or both source dimensions independently with probabilities *e*_ij_^Position^ and *e*_ij_^Color^, respectively. However, if item memory (1—*D*_ij_), bound source memory (1—*d*_ij_), and unbound source memory (1—*e*_ij_^Position^, 1—*e*_ij_^Color^) fail, participants are assumed to guess. In case of successful item memory but bound-source-memory and unbound-source-memory failure for either or both source dimensions, participants guess source A of source dimension *i* (e.g., “left” on source dimension “spatial position”) for a target item with probability *a*^Position^. They also guess source X of source dimension *j* (e.g., “blue” on source dimension “frame color”) for a target item assigned to source A (e.g., left) or B (e.g., right) of source dimension *i* (e.g., spatial position) with probability *a*_|left_^Color^ or *a*_|right_^Color^, respectively. If item memory fails, participants guess “old” with probability *b*. For unrecognized target or distractor items identified as “old,” participants guess source A of source dimension *i* (e.g., “left” on source dimension “spatial position”) with probability *g*^Position^. In addition, they guess source X of source dimension *j* (e.g., “blue” on source dimension “frame color”) for unrecognized target or distractor items assigned to source A (e.g., left) or B (e.g., right) of source dimension *i* (e.g., spatial position) with probability *g*_|left_^Color^ or *g*_|right_^Color^, respectively (Meiser, 2014).

In its most general version, the multidimensional source memory model allows for parameters that may differ between item types and sources, as illustrated in Fig. 3. To simplify this model and ensure identifiability of parameters, we employed basically the same principled strategy as previously used for the 2HTSM in Experiment 1. Specifically, we successively imposed the following constraints on the parameters (cf. Meiser, 2014; Meiser & Bröder, 2002): First, the item memory parameters *D*_ij_ were equated across the source dimensions “spatial position” and “frame color,” and *D*_New_ was constrained to be equal to the resulting item memory parameter *D*. Second, the bound source memory parameters *d*_ij_ were also equated across the source dimensions “spatial position” and “frame color” (parameter *d*). Next, the unbound source memory parameters for spatial position *e*_ij_^Position^ and frame color *e*_ij_^Color^ were equated across the source dimension “frame color” (parameter *e*^Position^) and “spatial position” (parameter *e*^Color^), respectively (Meiser, 2014; Meiser & Bröder, 2002). Finally, additional equality constraints were imposed on the source guessing parameters (i.e., *a*^Position^ = *g*^Position^, *a*_|left_^Color^ = *g*_|left_^Color^, *a*_|right_^Color^ = *g*_|right_^Color^).

Drawing on the active systems consolidation hypothesis, we predict for a 12-h retention interval that the corresponding item memory parameters, bound source memory parameters, and unbound source memory parameters *e*^Position^ and *e*^Color^ should be larger after sleep than wakefulness.

### Method

A 2 $\times$ 2 mixed factorial design with source dimension (spatial position vs. frame color) as within-subject factor and wake versus sleep as between-subjects factor was used in this experiment. As in Experiment 1, participants were randomly assigned to a wake or sleep condition and learned the material either in the morning or in the evening before they were tested following a 12-h retention interval.

#### Participants

To determine the necessary sample size for the model-based analysis a priori, we used multiTree (Moshagen, 2010). For an α-level of 0.05 and an assumed difference of 0.10 in the parameter of interest between the sleep and wake condition, the analysis for 130 participants, 120 target items, and 60 distractor items resulted in a power larger than 0.99 for item memory *D*, a power of 0.78 for bound source memory *d*, and power values of 0.67 and 0.61 for unbound source memories *e*^Position^ and *e*^Color^, respectively (for more detailed information, see the preregistration on the OSF, https://osf.io/a6z4u). As already detailed for Experiment 1, we aimed at a sample size of 130 participants in Experiment 2 and extended the pre-registered data collection period for the same reason as in Experiment 1. Specifically, we collected data from fall 2020 to spring 2021, using the same channels for participant recruitment as in Experiment 1. We made sure that participants of Experiment 1 did not additionally participate in Experiment 2 and vice versa. Furthermore, to participate in the experiment, participants had to be between 18 and 35 years old, speak German fluently, have no neurological disorders, and not be color blind (see the preregistration on the OSF, https://osf.io/a6z4u).

In total, 175 participants took part in the online experiment and were rewarded for successful completion with a flat fee of £6.00, as Experiment 2 took longer to complete than Experiment 1. Following the preregistered exclusion criteria, 14 participants were excluded because they indicated that they were distracted or interrupted during the experiment. One participant admitted not to have taken the testing session seriously and was thus excluded. Two further participants were excluded because the retention interval was not within 11–13 h. In addition, we excluded eight participants of the wake condition who napped during the retention interval. Another two participants reported having neurological disorders and were thus excluded. Furthermore, we excluded two participants because of substantial alcohol consumption during the retention interval, and three participants with larger false-alarm rates than hit rates.^7^ We also excluded nine additional participants for unforeseen reasons not included in the preregistration: Four participants reported technical problems, and one participant assigned to the wake condition delayed the start of the experiment so that it started at noon instead of the morning. Four additional participants were excluded because they indicated having detailed knowledge about the study design or the aim of the experiment. In sum, we excluded 41 participants, leaving 134 participants (*n*_wake_ = 62, *n*_sleep_ = 72) for analysis, all of them fluent in German. These 134 participants were between 18 and 35 years of age (*M* = 25.58 years, *SD* = 4.51), 84 (62.69%) were female. For all participants, many more than the minimally required 50% of the responses in the orienting task were correct (*M*_total_ = 97%, *M*_wake_ = 96%, *M*_sleep_ = 98%, see Table S4 in the OSM for more detailed sample characteristics), confirming that they paid attention to both source dimensions (i.e., spatial position and frame color) at encoding.

#### Materials

The stimulus material consisted of 200 grey-scaled object drawings selected from the multilingual picture databank (MultiPic; Duñabeitia et al., 2018). Of the 200 drawings, 120 target pictures were randomly chosen for each participant and displayed on either the left or the right side of the screen (i.e., 30 pictures each were displayed at the 10% and 90% position on the x-axis) with either a blue or a yellow colored frame (i.e., 30 pictures each were displayed in a blue colored 20-px frame with red–green–blue (RGB) values of 0, 40, 255, and in a complementary gold colored 20-px frame with RGB values of 255, 215, 0). Hence, spatial position (left vs. right) and frame color (blue vs. yellow) served as the two source dimensions of interest. Note that each source combination appeared equally often (i.e., 30 times). Another 60 pictures were randomly selected as distractors. Finally, four additional pictures were randomly selected as buffer items and presented in the beginning of the learning phase to prevent primacy effects. Hence, responses to these items were not included in our data analyses. As in Experiment 1, no recency buffer was included because of the 12-h retention interval. A list of the 200 pictures and detailed information about the selection criteria are available via the OSF (https://osf.io/8rmj2/?view_only=02e5eec5c3e54fd4aff3d55eedebffa7).

#### Procedure

The procedure of Experiment 2 (for an illustration, see Fig. 2) followed that of Experiment 1 and used the same online study builders (i.e., SoSci Survey, lab.js), but with the following changes to the source-monitoring task: During the study phase, 124 randomly selected pictures (i.e., four buffer and 120 target items) were sequentially presented on the left or right side of the screen in a blue or yellow colored frame for 5 s each with an interstimulus interval of 1 s (i.e., blank white screen for 500 ms followed by a fixation cross for 500 ms). The orienting task entailed pressing the correct button for spatial position and frame color of a picture shown on the screen. The two buttons for spatial position labeled “left” and “right” were arranged next to each other and were displayed in gray on the left side below the picture. The two buttons for frame color labeled “blue” and “yellow” were also arranged next to each other but displayed in the respective color on the right side below the picture. Only participants who answered with the correct combination for spatial position and frame color for more than 50% of the 124 pictures completed the learning session and were invited to the testing session 12 h later. Participants had to respond within the 5 s in which a picture was presented on the screen. They received no instruction about the order in which they should focus on the two source dimensions (i.e., spatial position, frame color). For the testing session, the 120 target items were intermixed with 60 distractor items and presented frameless in the middle of the screen with two buttons labeled “old” and “new” below. Note that the spatial position of the labels “old” and “new” was varied between participants as in Experiment 1. If participants answered “old,” they were asked about the spatial position and frame color of the picture. To respond, participants pressed one of the two respectively labeled left buttons for “left plus blue” or “left plus yellow” or one of the two respectively labeled right buttons for “right plus blue” or “right plus yellow.” Whereas the labels for spatial position were always presented on the respective sides of the screen, the position for the frame-color labels was varied between participants (i.e., for half of the participants the labels “left plus blue” and “right plus blue” were displayed above “left plus yellow” and “right plus yellow,” while this order was reversed for the other half of the participants; for a detailed description of the procedure, see the preregistration on the OSF, https://osf.io/a6z4u).

## Results

As in Experiment 1, we first analyzed effects of sleep versus wakefulness on commonly used measures of item and source memory. Again, we set a significance level of α = 0.05 for all analyses. Means, standard errors, and *t*-test results for hit and false alarm rates as well as *d’* and *c* of item recognition and ACSIM-based source memory for position and frame color are reported in Table 3.^8^ Concerning item memory, all two-tailed *t* tests between the sleep and wake groups were not statistically significant, *t*(132) ≤ 1.29, *p* ≥ 0.199 (see Table 3). To analyze ACSIM-based source memory, we performed a mixed ANOVA using source dimension (spatial position vs. frame color) as within-subject factor and wake versus sleep as between-subjects factor. There was a statistically significant main effect of the wake versus sleep condition, with better source memory after sleep (*M* = 0.61, *SE* = 0.01) than after wakefulness (*M* = 0.57, *SE* = 0.01), *F*(1, 132) = 6.71, *p* = 0.011, η_p_^2^ = 0.05. We also found a statistically significant main effect of source dimension, with better source memory for spatial position (*M* = 0.64, *SE* = 0.01) than for frame color (*M* = 0.55, *SE* = 0.01), *F*(1, 132) = 42.59, *p* < 0.001, η_p_^2^ = 0.24. However, there was no statistically significant interaction effect of the wake versus sleep condition with the source dimension, *F*(1, 132) = 2.97, *p* = 0.087. In sum, using commonly used item and source memory measures, we found a statistically significant sleep benefit in source memory but not in item memory.

**Table 3:** Results of item- and source-memory analyses in Experiment 2

Dependent variable	Wake		Sleep				
	*M*	*SE*	*M*	*SE*	*t*(132)	*p*	Cohen’s **d** [95% CI]
Item memory							
Hit rate	0.47	0.02	0.52	0.02	1.29	0.199	0.22 [-0.12, 0.56]
False-alarm rate	0.14	0.01	0.13	0.01	0.55	0.581	-0.08 [-0.42, 0.26]
Sensitivity index *d’*	1.16	0.07	1.32	0.07	1.25	0.212	0.21 [-0.13, 0.55]
Response bias *c*	0.66	0.04	0.60	0.04	0.72	0.474	-0.13 [-0.47, 0.21]
Source memory							
ACSIM, spatial position	0.60	0.01	0.67	0.01	2.86	0.005	0.56 [0.21, 0.90]
ACSIM, frame color	0.54	0.01	0.56	0.01	1.11	0.267	0.19 [-0.15, 0.53]

*Note*: Means and standard errors of the mean are shown for the wake (*n* = 62) and sleep condition (*n* = 72), as well as the results of two-tailed *t* tests comparing the two independent groups. We estimated Cohen’s **d** on the basis of the means and pooled standard deviations. Note that for both item and source memory, positive values of Cohen’s **d** indicate a sleep benefit, whereas negative values indicate a sleep disadvantage compared to wakefulness. ACSIM = average conditional source identification measure

In a second step, we tested our hypotheses using the MPT model of multidimensional source monitoring (Meiser, 2014) as described above. First, we fitted the most parsimonious model version, including equality constraints on the source guessing parameters (i.e., *a*^Position^ = *g*^Position^, *a*_|left_^Color^ = *g*_|left_^Color^, *a*_|right_^Color^ = *g*_|right_^Color^). Applying this model to the aggregated data, however, resulted in misfit, *G*^2^(24) = 41.42, *p* = 0.015. As in case of the 2HTSM used in Experiment 1, we therefore fitted the model without the additional constraints on the source guessing parameters to the aggregated data, which resulted in good fit, *G*^2^(18) = 23.82, *p* = 0.161. ML parameter estimates, standard errors, and 95% confidence intervals of this model version for the four experimental conditions are displayed in Table 4. We found a statistically significant difference between the sleep and wake condition in item memory *D*, Δ*G*^2^(1) = 31.54, *p* < 0.001, with about 6% larger item recognition estimates following sleep. This matches the result for item memory observed in the aggregate analyses of Experiment 1. By contrast, there was no statistically significant sleep benefit in bound source memory *d*, Δ*G*^2^(1) = 0.003, *p* = 0.955. The likely reason for this unexpected result is that in both the sleep and the wake condition the estimate of correctly identifying the source combination of recognized target items was very low (*d* = 0.04), reflecting a floor effect in episodic context-context binding. Because bound source memory is very low, the unbound source memory parameters *e*^Position^ and *e*^Color^ resemble the source memory parameter *d* for item-context binding of the 2HTSM in Experiment 1.^9^ Replicating Experiment 1, the unbound source memory parameter for spatial position *e*^Position^ differed significantly between the sleep and wake condition, Δ*G*^2^(1) = 7.93, *p* = 0.005. In line with the active systems consolidation hypothesis, the item-context binding probability for the source dimension “spatial position” was almost 13% larger in the sleep than in the wake condition. In contrast, there was no statistically significant sleep benefit for the unbound source memory parameter for color *e*^Color^, Δ*G*^2^(1) = 0.41, *p* = 0.523, although there was a descriptive pattern in the predicted direction. Similar to the result for bound source memory, it appears that this unexpected result is a consequence of the low probability of correctly identifying the source (i.e., blue, yellow) of recognized target items on the source dimension “frame color.” In fact, unbound source memory for color (*e*^Color^ ≤ 0.10) is significantly worse than for spatial position (*e*^Position^ ≥ 0.30) in both the sleep, Δ*G*^2^(1) = 135.92, *p* < 0.001, and the wake condition, Δ*G*^2^(1) = 46.98, *p* < 0.001. Concerning guessing parameters, we found no statistically significant differences between sleep and wake conditions whatsoever, all Δ*G*^2^(1) ≤ 2.19, *p* ≥ 0.139.

**Table 4:** Aggregated parameter estimates of the multinomial model of multidimensional source monitoring for Experiment 2

Parameter	Wake			Sleep		
	MLE	*SE*	95% CI	MLE	*SE*	95% CI
*D*	0.33	0.01	[0.32, 0.35]	0.40	0.01	[0.38, 0.41]
*d*	0.04	0.04	[0.00, 0.11]^a^	0.04	0.04	[0.00, 0.11]^a^
*e*^Position^	0.30	0.04	[0.23, 0.37]	0.43	0.03	[0.37, 0.49]
*e*^Color^	0.07	0.04	[0.00, 0.15]^a^	0.10	0.04	[0.02, 0.18]
*a*^Position^	0.48	0.02	[0.44, 0.52]	0.43	0.02	[0.39, 0.47]
*a*_|left_^Color^	0.51	0.02	[0.46, 0.55]	0.50	0.02	[0.46, 0.53]
*a*_|right_^Color^	0.61	0.03	[0.54, 0.67]	0.57	0.03	[0.51, 0.63]
*b*	0.21	0.01	[0.19, 0.22]	0.21	0.01	[0.19, 0.22]
*g*^Position^	0.51	0.02	[0.46, 0.55]	0.55	0.02	[0.50, 0.59]
*g*_|left_^Color^	0.49	0.03	[0.43, 0.55]	0.53	0.03	[0.48, 0.58]
*g*_|right_^Color^	0.47	0.03	[0.40, 0.53]	0.52	0.03	[0.45, 0.58]

*Note*: For the aggregated model-based analysis, maximum likelihood estimates (MLE), standard errors (*SE*), and 95% confidence intervals (CI) are reported. *D* = probability of correctly identifying a target item as “old” and a distractor item as “new”; *d* = probability of correctly identifying the source combination of a target item; *e*^Position^ = probability of correctly identifying the source (i.e., left, right) on source dimension “spatial position” if the target item was correctly identified as “old”; *e*^Color^ = probability of correctly identifying the source (i.e., blue, yellow) on source dimension “frame color” if the target item was correctly identified as “old”; *a*^Position^ = probability of guessing ‘‘left” on source dimension “spatial position” if the target item was correctly identified as “old”; *a*_|left_^Color^ = probability of guessing “blue” on source dimension “frame color” if the target item was correctly identified as “old” and assigned to source “left”; *a*_|right_^Color^ = probability of guessing “blue” on source dimension “frame color” if the target item was correctly identified as “old” and assigned to source “right”; *b* = probability of guessing that an item is “old”; *g*^Position^ = probability of guessing “left” on source dimension “spatial position” if the unrecognized item was guessed to be “old”; *g*_|left_^Color^ = probability of guessing “blue” on source dimension “frame color” if the unrecognized item was guessed to be “old” and assigned to source “left”; *g*_|right_^Color^ = probability of guessing “blue” on source dimension “frame color” if the unrecognized item was guessed to be “old” and assigned to source “right”

^a^Asymptotic CI boundaries with values below 0.00 or above 1.00 were set to 0 and 1, respectively, because the parameter space limits the range of admissible values to [0.00, 1.00]

As in Experiment 1, we checked the robustness of our results by performing a hierarchical model-based analysis using Klauer’s (2010) latent-trait model as implemented in TreeBUGS (Heck et al., 2018) for partial pooling. Again, as detailed in the Appendix (see Table 7), the estimated group-level means of the hierarchical MPT model closely resembled those reported in Table 4. We conclude that the basic result pattern is robust against using complete versus partial pooling data analysis methods.

## Discussion

Replicating Experiment 1 and some of the previous studies (e.g., Mawdsley et al., 2014; van der Helm et al., 2011), we observed a significant sleep benefit in ACSIM-based and model-based source memory for spatial position in Experiment 2. This supports a core prediction of the active systems consolidation hypothesis, namely, that sleep compared to wakefulness should benefit item-context-binding in episodic memory for retention intervals up to 12 h. However, this core prediction was not confirmed for the second source dimension “frame color,” that is, the corresponding item-context binding parameter, *e*^Color^, did not differ significantly between sleep and wake conditions. Moreover, compared to source dimension “spatial position,” source dimension “frame color” exhibited considerably worse source memory in both sleep and wake conditions, suggesting that frame colors were only weakly encoded.

Although some empirical findings suggest that weakly encoded memories profit more from memory consolidation during sleep than stronger encoded memories (e.g., Denis et al., 2020; Drosopoulos et al., 2007), there are other studies showing that stronger memories benefit more (e.g., Schoch et al., 2017; Tucker & Fishbein, 2008). At first glance, these results appear contradictory. They are, however, in line with the assumption that sleep benefits follow an inverted U-shaped function of memory strength (Stickgold, 2009). According to this account, sleep benefits increase with encoding strength up to a medium level of memory strength before they decrease. In Experiment 2, memory strength likely varies within the lower limb of the inverted U-shaped function only, as indicated by the unbound source memory parameter estimates of the aggregated model-based analysis for spatial position (*e*^Position^ ≤ 0.43) and frame color (*e*^Color^ ≤ 0.10). Thus, the consolidation-based sleep benefit in source memory should be larger for spatial position than for frame color. However, for frame color, we observed no significant sleep benefit in source memory. This result can be explained by previous research showing that a minimum level of memory strength at encoding is necessary for the sleep benefit to occur (e.g., Denis et al., 2020; Muehlroth et al., 2020; Rauchs et al., 2011). The non-significant sleep benefit in source memory for frame color may therefore be a consequence of insufficient encoding.

Former studies that used color as source dimension to investigate episodic context-binding enforced intentional learning of item-context associations (e.g., Köster et al., 2017; Wang & Fu, 2009). In contrast, item-context and context-context associations were learned incidentally in our Experiment 2 to create a more realistic setting that resembles everyday source monitoring. Obviously, although we employed an orienting task towards both source dimensions, incidental learning does not seem to support encoding of the frame color context.

In fact, frame color appears to be less salient as a context feature than spatial position. For at least two reasons, an item’s frame color is less likely to be encoded successfully than its spatial position. First, from an evolutionary perspective, it has often been argued that spatial position is more important for survival and thus receives prioritized processing (Hasher & Zacks, 1979; Nairne et al., 2012; Yin et al., 2019). Second, according to the object-file theory of visual perception (Kahneman et al., 1992; Mitroff et al., 2004, 2005), the spatial position of an object is encoded in a first step by default. Other details such as color are added in a second step that requires more elaborated processing (Chen & Wyble, 2015). Taking these theories into account, it does not come as a surprise that context memory for frame color is considerably worse than context memory for spatial position, irrespective of sleep or wake states during retention.

In terms of item memory, Experiment 2 replicated the mixed results of Experiment 1 and previous research (e.g., Mawdsley et al., 2014; van der Helm et al., 2011). Like in Experiment 1, the observed descriptive result patterns of *d’* and the aggregated as well as hierarchical model-based analyses suggest that sleep compared to wakefulness might benefit item recognition. Again, this pattern was significant only for complete pooling. Hence, the results of Experiment 1 and Experiment 2 might hint at a sleep benefit in item recognition, which is, however, hard to detect due to its small size. In an attempt to remedy this problem by increasing statistical power, we reanalyzed both the sensitivity index *d’* and the response bias *c* based on the combined data of Experiment 1 and Experiment 2 (*N* = 266, *n*_wake_ = 127, *n*_sleep_ = 139). To analyze item memory, we performed a between-subjects ANOVA using the condition (wake vs. sleep) and the study (Experiment 1 vs. Experiment 2) as between-subjects factors. For *d’* as well as* c*, there was a statistically significant main effect of the study, with better discrimination between target and distractor items and a weaker “new”-response bias in Experiment 1 (*d’*: *M* = 2.16, *SE* = 0.06; *c*: *M* = 0.42, *SE* = 0.02) than in Experiment 2 (*d’*: *M* = 1.24, *SE* = 0.05; *c*: *M* = 0.63, *SE* = 0.03), *d’*: *F*(1, 262) = 77.47, *p* < 0.001, η_p_^2^ = 0.23; *c*: *F*(1, 262) = 16.37, *p* < 0.001, η_p_^2^ = 0.06. By contrast, despite the larger overall sample size, there was still no statistically significant main effect of wake (*d’*: *M* = 1.61, *SE* = 0.06; *c*: *M* = 0.53, *SE* = 0.02) versus sleep (*d’*: *M* = 1.78, *SE* = 0.06; *c*: *M* = 0.52, *SE* = 0.02), *d’*: *F*(1, 262) = 3.38, *p* = 0.067, η_p_^2^ = 0.01; *c*: *F*(1, 262) = 0.13, *p* = 0.720, η_p_^2^ = 0.00, and also no statistically significant interaction effect of the study and the wake versus sleep condition, *d’*: *F*(1, 262) = 0.07, *p* = 0.795, η_p_^2^ = 0.00; *c*: *F*(1, 262) = 0.56, *p* = 0.454, η_p_^2^ = 0.00. A possible reason is that even the combined *N* = 266 of both experiments does not provide sufficient power to detect relatively small sleep benefits in item recognition memory. In fact, according to an a priori power analysis for a two-tailed *t* test with two independent groups, an error probability α of 0.05, and a target-power of 1—β = 0.95, it would require a total sample size of 362 participants (Faul et al., 2007) to detect a sleep benefit of size Hedges’ *g* = 0.38 for recognition tasks, as reported in the meta-analysis of Berres and Erdfelder (2021).

In sum, Experiment 2 confirms the conclusion drawn from Experiment 1 that sleep improves item-context binding of salient features such as spatial position across a 12-h retention interval, in line with the active systems consolidation hypothesis. In addition to this successful conceptual replication, Experiment 2 also explored whether sleep improves context-context binding. However, bound source memory for spatial position and frame color did not differ significantly between sleep and wake conditions, most likely because of floor effects in either condition. Combined with the non-significant sleep benefit in source memory for the less salient source dimension “frame color,” this result suggests that a sufficiently high memory strength of item-context and context-context associations at encoding is necessary for the sleep benefit to occur. Whereas spatial position appears to be a context feature that receives sufficient processing during encoding, frame color apparently does not – at least under the incidental learning conditions employed in Experiment 2.

## General discussion

In two experiments, we tested a core assumption of the active systems consolidation hypothesis, namely, that sleep benefits context-binding in episodic memory for relatively short retention intervals of up to 12 h. In contrast to previous research, we made use of MPT models that provide uncontaminated measures of source memory. Both experiments consistently showed a sleep benefit in source memory for spatial position as predicted by the active systems consolidation account. In contrast, the results for item memory were mixed, a results pattern that is in line with previous research.

Using MPT models to decompose source-monitoring performance in effects of separate underlying cognitive processes is a powerful alternative to traditional measures. However, there are also caveats that must be considered when using MPT models. Specifically, by imposing equality constraints on model parameters of source monitoring MPT models, several submodels can be defined. This raises the problem of determining which model to use (Bayen et al., 1996). Usually, the most parsimonious model that still fits the data is selected (i.e., the model with the smallest number of free parameters). In case of the 2HTSM, the most parsimonious model is Submodel 4 with four parameters only. This submodel imposes equality constraints on all item memory parameters, all source memory parameters, and on the guessing parameters *a* and *g*, respectively (Bayen et al., 1996). However, we were forced to relax the equality constraint for the guessing parameters (leading to Submodel 5a with 5 free parameters), because applying Submodel 4 to the aggregated data of Experiment 1 resulted in misfit (see the *Results* section of Experiment 1). Alternatively, one could also relax the equality constraint for the source memory parameters instead of the two source guessing parameters, resulting in a data equivalent model (i.e., Submodel 5d of Bayen et al., 1996) that fits the data as well as Submodel 5a. Most important with respect to our research questions, however, both Submodel 5a and Submodel 5d showed statistically significant sleep benefits in item memory and in source memory for at least one of the two sources involved.^10^ In other words, our substantive conclusions concerning sleep benefits in item and source memory would not be affected by whether we prefer Submodel 5a or 5d for data analysis.

Fortunately, the more complex design of Experiment 2 with two source dimensions circumvents problems of equivalent submodels (Bröder & Meiser, 2007). The corresponding multidimensional source monitoring model clearly showed that sleep improves unbound source memory for both spatial positions (i.e., left and right) to the same degree. Taking this into account, it seems safe to adopt a model with a single source memory parameter (i.e., Submodel 5a) also for Experiment 1. Most importantly, however, all model-based results provide unequivocal evidence for a sleep benefit in episodic memory binding of spatial context features, irrespective of the 2HTSM submodel used.

Aside from the measures used to assess item and source memory, there are further potential moderators that may have contributed to the mixed results in previous research, specifically, the sleep study design employed and the encoding strength of relevant episodic information. As already outlined in the *Introduction*, researchers have used different sleep study designs to investigate sleep benefits in episodic memory context-binding, ranging from split-night designs (e.g., Groch et al., 2015; Rauchs et al., 2004; Sopp et al., 2017), naps (e.g., Köster et al., 2017; van der Helm et al., 2011; Wang & Fu, 2009), to comparisons of natural night sleep with daytime wakefulness (e.g., Lewis et al., 2011; Mawdsley et al., 2014; Wang et al., 2017). All these sleep studies differ in the amount of SWS associated with sleep, a sleep feature assumed to be essential for memory consolidation (e.g., Klinzing et al., 2019; Lewis & Durrant, 2011). In fact, a recent meta-analysis on the sleep benefit in episodic memory showed that sleep benefits tend to be larger for sleep study designs associated with large amounts of SWS (Berres & Erdfelder, 2021). It is therefore likely that different sleep study designs additionally contributed to the mixed results reported in the literature.

Weak encoding strength may explain why we did not observe significant sleep benefits in source memory for frame color and in bound source memory for spatial position and frame color. The fact that the corresponding MPT parameters were at or near floor level suggests that sufficiently high memory strength of item-context and context-context associations at encoding is necessary for the sleep benefit to occur (cf. Denis et al., 2020; Muehlroth et al., 2020; Rauchs et al., 2011). The strength of memory representations is affected by various aspects of the encoding situation, such as presentation time, scope and type of the stimulus material, and encoding instructions. In fact, whereas the current experiments and few others employed incidental learning of item-context associations (e.g., Mawdsley et al., 2014; Wang et al., 2017), most previous studies used intentional learning, in some cases even with the explicit instruction to use mnemonic strategies that include context encoding (e.g., Köster et al., 2017; Lewis et al., 2011). Moreover, different source dimensions require different degrees of cognitive effort to be encoded successfully (see the *Discussion* section of Experiment 2). These aspects, among others, may have affected the strength of the memory representation at encoding, contributing to the mixed results in different sleep studies of source monitoring found thus far.

In line with the active systems consolidation hypothesis, we found convincing evidence for a sleep benefit in source memory using a 12-h retention interval. Note, however, that sleep benefits in episodic memory can be explained not only by memory consolidation but also by reduced retroactive interference (for a review of consolidation and interference theories, see Berres & Erdfelder, 2021). The contextual binding account (Yonelinas et al., 2019), for example, explains the sleep benefit in terms of a passive effect on memory retrieval. Specifically, retrieval of a target information can be impaired by information learned before or after, provided the content or context of the interfering and the target information resemble each other. In other words, context similarity (i.e., similarity of any aspect of a specific learning situation such as spatial position or color) may foster retroactive interference. During sleep, however, new learning is virtually absent. Thus, retroactive interference due to content or context similarity is reduced which in turn should facilitate retrieval of the target information after sleep compared to wakefulness (Yonelinas et al., 2019).

Yet, in terms of source memory, sleep benefits due to reduced retroactive interference appear to play a minor role. According to the memory-system dependent forgetting hypothesis (Hardt et al., 2013; see also Sadeh et al., 2014), interference effects on hippocampal memory representations, such as item-item or item-context associations, should be “minimal” (Hardt et al., 2013, p. 111). As such, the circuit architecture of the hippocampus is assumed to allow efficient pattern separation by assigning orthogonal representations even to highly similar information, thereby diminishing overlapping neuronal populations and thus interference. By contrast, memory representations of item memory, linked to extrahippocampal regions, are represented by overlapping neuronal populations. As a consequence, these memories should be very susceptible to interference (Hardt et al., 2013). Indeed, supporting this theory, Kuhlmann et al. (2021) investigated forgetting over short, interference-filled lags in three experiments and found pronounced interference-based forgetting in item memory compared to item-item and item-context associative memory (see also Sadeh et al., 2014).

Taking the memory-system dependent forgetting hypothesis into account when considering underlying processes of the sleep benefit in source memory, two assumptions can be made: First, because interference effects should be minimal for source memory, it can be assumed that sleep benefits in item-item and item-context associative memory depend more on memory consolidation than on reduced retroactive interference. Therefore, we interpret our results for source memory in terms of the active systems consolidation hypothesis, although additional sleep benefits on memory retrieval as predicted by the contextual binding account cannot be ruled out completely.

Second, for item memory, which should be very susceptible to interference, it can be assumed that sleep benefits are more heavily based on retrieval advantages due to reduced retroactive interference compared to source memory. This assumption can also offer a possible explanation for the mixed evidence of sleep benefits in item memory. To reiterate, the results of Experiment 1 and Experiment 2 might hint to a small sleep benefit in item recognition. The small size of the effect might be due to the fact that we assessed item memory with a recognition task. Recent meta-analyses suggest that the sleep benefit is moderated by the retrieval procedure (e.g., Berres & Erdfelder, 2021; Newbury & Monaghan, 2019). Consider for example the meta-analysis by Berres and Erdfelder (2021) who observed the largest sleep benefit in free recall (Hedges’ *g* = 0.49), followed by cued recall (Hedges’ *g* = 0.45), and lastly recognition tasks (Hedges’ *g* = 0.38). Correspondingly, Newbury and Monaghan (2019) observed better memory for recall (Hedges’ *g* = 0.41) than for recognition (Hedges’ *g* = 0.01), although the sleep benefit was not statistically significant for either task. As such, free recall relies more heavily on memory retrieval than cued recall, and cued recall more than recognition. Thus, sleep benefits due to reduced retroactive interference should be largest in free recall, followed by cued recall, and lastly recognition tasks (cf. Dyne et al., 1990; McKinney, 1935; Postman, 1952). This might explain why findings regarding sleep benefits in item memory are mixed.

Further research is needed to investigate the contribution of encoding, storage, and retrieval to sleep benefits in item and source memory. In the current experiments we employed incidental learning of item-context associations and therefore did not measure immediate memory performance. However, to disentangle these processes, it is necessary to record memory performance not only in a delayed but also in an immediate memory test. Again, appropriately designed MPT models (cf. Bröder, 2009; Erdfelder et al., 2024; Symeonidou & Kuhlmann, 2021) could provide additional insights alongside typical change measures of immediate and delayed test performance.

Apart from the processes underlying the sleep benefit in item and source memory, several open questions remain for future research. First, probably because bound source memory parameter *d* was at floor level (Experiment 2), no significant sleep benefit in context-context binding emerged. Future studies should therefore ensure that context-context associations are encoded with sufficient memory strength to allow for a rigorous test of the hypothesis that sleep benefits bound source memory. This may, however, require switching to intentional learning instructions and associative encoding strategies and thus a very specific type of encoding only.

Second, in the current experiments, we investigated the sleep benefit in source memory using a 12-h retention interval. However, the precise time course of memory consolidation during sleep is not yet well understood (Dudai, 2004, 2012; Dudai et al., 2015; Klinzing et al., 2019; Lewis & Durrant, 2011; Pöhlchen & Schönauer, 2020; Stickgold, 2005; Stickgold & Walker, 2007). In our experiments, sleep duration and sleep quality were assessed via self-report only. Therefore, further studies using standardized designs with different retention intervals, combined with objective measures of sleep quality obtained via polysomnography, are necessary to explore the minimum and the maximum length of retention intervals for which sleep improves source memory in more detail, including the associated sleep-specific features (e.g., sleep spindles) that may moderate this improvement. Further, such objective measures may also reveal subtle sleep benefits in item recognition.

Third, context-recollection in episodic memory is often not only assessed with source memory measures but also with remember-know judgments to capture subjective retrieval experiences with respect to conscious recollection and familiarity in addition (for a review, see Inostroza & Born, 2013). In the current experiments, we used source memory measures only because they reflect hippocampus-dependent memories more directly than remember-know judgments. Nevertheless, extending model-based analyses to include remember-know judgments (see, e.g., Meiser, 2014) may provide more fine-graded insights into sleep-dependent benefits in episodic memory context-binding. In this context it is particularly interesting to investigate less hippocampus-dependent memories in future research, as recent studies suggest that memories which likely do not require the hippocampus during encoding (e.g., memory for motor sequences) may nevertheless depend on it for consolidation during sleep (e.g., Sawangjit et al., 2018; Schapiro et al., 2019).

## Conclusion

Our two experiments consistently show that sleep benefits source memory, provided that relevant context features – such as the spatial position of an item – are sufficiently salient and thus well encoded. These results are in line with the prediction of the active systems consolidation hypothesis that sleep benefits item-context-binding for retention intervals of about 12 h. In addition, our findings call attention to potential moderators that may explain the mixed results in previous research, such as level of analysis employed or the encoding strength of source dimensions that prevents sleep benefits in context-context bindings when at least one source dimension is insufficiently encoded (see Experiment 2). In sum, the present research adds to the growing empirical evidence that memory consolidation as described by the active systems consolidation hypothesis is one of the key neurocognitive processes that contributes to the sleep benefit in episodic memory.

## Electronic supplementary material

Below is the link to the electronic supplementary material.

Supplementary file1 (DOCX 52 KB)

## Data Availability

Hypotheses, study designs, sample sizes, and analysis plans were preregistered (Experiment 1: https://osf.io/gctzn; Experiment 2: https://osf.io/a6z4u). Additional materials (i.e., list of stimuli, codebook, data set) are provided online on the Open Science Framework (OSF; https://osf.io/8rmj2/?view_only=02e5eec5c3e54fd4aff3d55eedebffa7).

## Appendix A

**Table 5:** Study characteristics of previous research on sleep benefits in source memory

References	*N*	Age	Experimental design	RI	Stimulus material	Encoding instruction	Delayed memory test	Memory measure	Sleep benefit Cohen’s **d** [95% CI] ^b^
Split-night designs									
Groch et al. (2015); Experiment 1, neutral memories	18	21.27	Within-subjects	3.00					
Item, SWS rich early sleep, retention Item, REM rich late sleep, retention Source, SWS rich early sleep, retention Source, REM rich late sleep, retention Source, SWS rich early sleep, retention Source, REM rich late sleep, retention					Pictures Pictures Frame color Frame color Location Location	Intentional Intentional Intentional Intentional Intentional Intentional	Recognition Recognition Cued recall Cued recall Cued recall Cued recall	Hits–false alarms Hits–false alarms Hits Hits Hits Hits	— — — — — —
Groch et al. (2015); Experiment 2, neutral memories	18	22.11	Within-subjects	3.00					
Item, SWS rich early sleep, retention Item, REM rich late sleep, retention Source, SWS rich early sleep, retention Source, REM rich late sleep, retention					Pictures Pictures Frame color Frame color	Intentional Intentional Intentional Intentional	Recognition Recognition Cued recall Cued recall	Hits–false alarms Hits–false alarms Hits Hits	— — — —
Rauchs et al. (2004); what-where-when task^a^	43	20.18	Between-subjects	4.25					
Item, SWS rich early sleep, “what” Item, REM rich late sleep, “what” Item, SWS rich early sleep, “what,” change Item, REM rich late sleep, “what,” change Item, SWS rich early sleep, “what” Item, REM rich late sleep, “what” Item, SWS rich early sleep, “what,” remember Item, REM rich late sleep, “what,” remember Source, SWS rich early sleep, “where” Source, REM rich late sleep, “where” Source, SWS rich early sleep, “where,” change Source, REM rich late sleep, “where,” change Source, SWS rich early sleep, “where” Source, REM rich late sleep, “where” Source, SWS rich early sleep, “where,” remember Source, REM rich late sleep, “where,” remember Source, SWS rich early sleep, “when” Source, REM rich late sleep, “when” Source, SWS rich early sleep, “when,” change Source, REM rich late sleep, “when,” change Source, SWS rich early sleep, “when” Source, REM rich late sleep, “when” Source, SWS rich early sleep, “when,” remember Source, REM rich late sleep, “when,” remember					Words Words Words Words Words Words Words Words Location Location Location Location Location Location Location Location Word list Word list Word list Word list Word list Word list Word list Word list	Intentional Intentional Intentional Intentional Intentional Intentional Intentional Intentional Intentional Intentional Intentional Intentional Intentional Intentional Intentional Intentional Intentional Intentional Intentional Intentional Intentional Intentional Intentional Intentional	Free recall Free recall Free recall Free recall Recognition Recognition Recognition Recognition Free recall Free recall Free recall Free recall Cued recall Cued recall Cued recall Cued recall Free recall Free recall Free recall Free recall Cued recall Cued recall Cued recall Cued recall	Hits Hits Hits Hits Hits Hits Hits Hits ACSIM ACSIM ACSIM ACSIM ACSIM ACSIM ACSIM ACSIM ACSIM ACSIM ACSIM ACSIM ACSIM ACSIM ACSIM ACSIM	0.57 [-0.26, 1.41] 0.27 [-0.62, 1.15] 0.60 [-0.24, 1.43] 0.19 [-0.70, 1.07] 0.65 [-0.19, 1.49] 0.56 [-0.34, 1.46] 0.33 [-0.49, 1.15] -0.13 [-1.01, 0.75] -0.71 [-1.55, 0.13] 1.09 [0.14, 2.03] 0.08 [-0.74, 0.90] 0.84 [-0.08, 1.76] 0.50 [-0.33, 1.33] 0.10 [-0.79, 0.98] -0.38 [-1.20, 0.45] 0.38 [-0.50, 1.27] -0.03 [-0.85, 0.79] 0.26 [-0.63, 1.14] 0.60 [-0.24, 1.43] 0.44 [-0.45, 1.33] 0.72 [-0.12, 1.57] -0.02 [-0.90, 0.86] 0.25 [-0.57, 1.07] 0.39 [-0.50, 1.28]
Sopp et al. (2017); neutral memories	38	22.74	Between-subjects	3.00					
Item, SWS rich early sleep Item, REM rich late sleep Source, SWS rich early sleep Source, REM rich late sleep					Pictures Pictures Location Location	Intentional Intentional Intentional Intentional	Recognition Recognition Cued recall Cued recall	Hits–false alarms Hits–false alarms ACSIM ACSIM	— — — —
Nap designs									
Köster et al., 2017; Night-time nap	26	20.00	Between-subjects	6.00					
Item Source					Pictures Color	Intentional Intentional	Recognition Cued recall	Hits–false alarms Hits–false alarms	0.40 [-0.38, 1.18] 0.00 [-0.77, 0.77]
Lewis et al., 2011; Experiment 2, daytime nap	38	20.92	Between-subjects	5.00					
Item, emotion task Source, emotion task, neutral Source, emotion task, neutral, change score					Pictures Pictures Pictures	Intentional Intentional Intentional	Recognition Cued recall Cued recall	Hits Hits Hits	0.35 [-0.29, 0.99] 0.58 [-0.06, 1.23] 0.60 [-0.05, 1.25]
van der Helm et al., 2011; daytime nap	27	20.60	Between-subjects	6.00					
Item Source					Words Poster	Intentional Intentional	Recognition Cued recall	*d’* SIM	0.11 [-0.64, 0.87] 0.98 [0.18, 1.78]
Wang & Fu, 2009; daytime nap	40	21.54	Between-subjects	2.00					
Item, male Item, female Source, male Source, female Source, male, change score ^a^ Source, female, change score ^a^					Picture Picture Color Color Color Color	Intentional Intentional Intentional Intentional Intentional Intentional	Recognition Recognition Cued recall Cued recall Cued recall Cued recall	Hits–false alarms Hits–false alarms ACSIM ACSIM ACSIM ACSIM	-0.17 [-1.04, 0.71] 0.37 [-0.52, 1.25] -1.05 [-1.99, -0.12] 1.17 [0.22, 2.11] -1.53 [-2.52, -0.53] 1.32 [0.35, 2.28]
Natural sleep and wakefulness designs									
Lewis et al., 2011; Experiment 1	22	24.50	Between-subjects	12.00					
Item, emotion task Item, people task Source, emotion task, neutral Source, people task, neutral Source, combined data, neutral, change score					Pictures Pictures Pictures Pictures Pictures	Intentional Intentional Intentional Intentional Intentional	Recognition Recognition Cued recall Cued recall Cued recall	Hits Hits Hits Hits Hits	0.55 [-0.30, 1.40] 0.38 [-0.47, 1.22] 0.61 [-0.25, 1.46] 0.36 [-0.48, 1.20] 0.78 [-0.08, 1.65]
Mawdsley et al., 2014^a^	40	40.30	Between-subjects	12.00					
Item, day workers Item, shift workers Source, day workers Source, shift workers					Words Words Location Location	Incidental Incidental Incidental Incidental	Recognition Recognition Cued recall Cued recall	Hits–false alarms Hits–false alarms Hits Hits	1.43 [0.45, 2.41] 2.78 [1.55, 4.00] 1.74 [0.71, 2.77] 2.75 [1.52, 3.97]
Wang et al., 2017; children	38	9.99	Between-subjects	11.00					
Item-item, retention ^a^ Source Source, retention ^a^ Source, correct item-item association, retention ^a^					Word pairs Word list Word list Word list	Intentional Incidental Incidental Incidental	Cued recall Cued recall Cued recall Cued recall	Hits Hits Hits Hits	0.84 [0.14, 1.53] 0.60 [-0.08, 1.29] 0.92 [0.22, 1.62] 0.44 [-0.24, 1.12]

Cohen’s **d **was estimated on the basis of the reported means and standard deviations or standard errors. Note that positive and negative Cohen’s **d**-values indicate a sleep benefit and a sleep disadvantage compared to wakefulness, respectively. For Groch et al. (2015) and Sopp et al. (2017) no Cohen’s **d** was computed as the reported experiments either do not include a wake condition or fail to report the necessary information. Age is reported in years based on available information about the mean age of participants included in the analysis of the respective study.

RI = retention interval in hours; SWS = slow-wave sleep; REM = rapid eye movement; retention = delayed test performance in percent with immediate test performance set to 100%; change = difference between immediate and delayed test performance; remember = remember judgments in remember-know paradigms; daytime nap = participants in the sleep condition nap before 7 p.m., night-time nap = participants in the sleep condition nap after 7 p.m.; *d’* = sensitivity index of the signal detection theory; SIM = source identification measure; ACSIM = average conditional source identification measure.

^a^ We used the open-source WebPlotDigitizer (Rohatgi, 2022) to obtain the necessary values for calculating Cohen’s **d**.

^b^ The sleep benefit effect size estimates (Cohen’s **d**) and the respective 95% confidence intervals were calculated with the web-based effect size calculator provided by David B. Wilson (https://www.campbellcollaboration.org/escalc/html/EffectSizeCalculator-Home.php; Lipsey & Wilson, 2001)

## Appendix B

### Results of the Bayesian-hierarchical multinomial processing tree (MPT) model analyses

**Table 6:** Hierarchical Bayesian parameter estimates of the latent-trait version of the two-high-threshold multinomial model of source monitoring (2HTSM) for Experiment 1

Parameter	Wake			Sleep		
	*M*	*SD*	95% BCI	*M*	*SD*	95% BCI
*D*	0.62	0.03	[0.55, 0.68]	0.68	0.03	[0.62, 0.73]
*d*	0.42	0.05	[0.33, 0.51]	0.62	0.04	[0.54, 0.69]
*a*	0.44	0.03	[0.37, 0.51]	0.48	0.03	[0.42, 0.53]
*b*	0.18	0.03	[0.13, 0.24]	0.17	0.03	[0.11, 0.23]
*g*	0.49	0.05	[0.40, 0.58]	0.70	0.08	[0.53, 0.86]

For the latent-trait model (Klauer, 2010), posterior means (*M*), posterior standard deviations (*SD*), and 95% Bayesian credibility intervals (BCI) of the probability transformed group-level parameters as estimated with TreeBUGS (Heck et al., 2018) are reported. There was good MCMC chain convergence ($\widehat{\text{R}}$< 1.05) and model fit (wake condition: *p*_T1_ = 0.39, *p*_T2_ = 0.50; sleep condition: *p*_T1_ = 0.58, *p*_T2_ = 0.41). *D* = probability of correctly identifying a target item as “old” and a distractor item as “new”; *d* = probability of correctly identifying the target item source; *a* = probability of guessing that a correctly identified target item is from source “left”; *b* = probability of guessing that an item is “old”; *g* = probability of guessing that an unrecognized item is from source “left” if it was guessed to be “old”; $\widehat{\text{R}}$= potential scale reduction factor (Gelman & Rubin, 1992); *p*_T1_ = posterior predictive *p*-value for the mean; *p*_T2_ = posterior predictive *p*-value for the covariance

**Table 7:** Hierarchical Bayesian parameter estimates of the latent-trait version of the multinomial model of multidimensional source monitoring for Experiment 2

Parameter	Wake			Sleep		
	*M*	*SD*	95% BCI	*M*	*SD*	95% BCI
*D*	0.29	0.03	[0.23, 0.36]	0.36	0.03	[0.31, 0.42]
*d*	0.01	0.01	[0.00, 0.03]	0.01	0.01	[0.00, 0.04]
*e* ^Position^	0.21	0.05	[0.10, 0.31]	0.34	0.06	[0.22, 0.46]
*e* ^Color^	0.01	0.01	[0.00, 0.04]	0.01	0.01	[0.00, 0.03]
*a* ^Position^	0.44	0.03	[0.38, 0.50]	0.45	0.03	[0.40, 0.51]
*a*_|left_^Color^	0.54	0.04	[0.46, 0.61]	0.52	0.02	[0.47, 0.56]
*a*_|right_^Color^	0.63	0.05	[0.54, 0.75]	0.58	0.05	[0.48, 0.68]
*b*	0.17	0.02	[0.13, 0.22]	0.19	0.02	[0.15, 0.23]
*g* ^Position^	0.53	0.03	[0.47, 0.58]	0.53	0.03	[0.47, 0.59]
*g*_|left_^Color^	0.46	0.04	[0.39, 0.53]	0.48	0.04	[0.40, 0.56]
*g*_|right_^Color^	0.47	0.03	[0.40, 0.54]	0.51	0.04	[0.44, 0.59]

For the latent-trait model (Klauer, 2010), posterior means (*M*), posterior standard deviations (*SD*), and 95% Bayesian credibility intervals (BCI) of the probability transformed group-level parameters as estimated with TreeBUGS (Heck et al., 2018) are reported. There was good MCMC chain convergence ($\widehat{\text{R}}$< 1.05) and model fit (wake condition: *p*_T1_ = 0.52, *p*_T2_ = 0.42; sleep condition: *p*_T1_ = 0.05, *p*_T2_ = 0.48). *D* = probability of correctly identifying a target item as “old” and a distractor item as “new”; *d* = probability of correctly identifying the source combination of a target item; *e*^Position^ = probability of correctly identifying the source (i.e., left, right) on source dimension “spatial position” independent from source dimension “frame color” if the target item was correctly identified as “old”; *e*^Color^ = probability of correctly identifying the source (i.e., blue, yellow) on source dimension “frame color” independent from source dimension “spatial position” if the target item was correctly identified as “old”; *a*^Position^ = probability of guessing ‘‘left” on source dimension “spatial position” if the target item was correctly identified as “old”; *a*_|left_^Color^ = probability of guessing “blue” on source dimension “frame color” if the target item was correctly identified as “old” and assigned to source “left”; *a*_|right_^Color^ = probability of guessing “blue” on source dimension “frame color” if the target item was correctly identified as “old” and assigned to source “right”; *b* = probability of guessing that an item is “old”; *g*^Position^ = probability of guessing “left” on source dimension “spatial position” if the unrecognized item was guessed to be “old”; *g*_|left_^Color^ = probability of guessing “blue” on source dimension “frame color” if the unrecognized item was guessed to be “old” and assigned to source “left”; *g*_|right_^Color^ = probability of guessing “blue” on source dimension “frame color” if the unrecognized item was guessed to be “old” and assigned to source “right”; $\widehat{\text{R}}$= potential scale reduction factor (Gelman & Rubin, 1992); *p*_T1_ = posterior predictive *p*-value for the mean; *p*_T2_ = posterior predictive *p*-value for the covariance

## Footnotes

1 We use the terms source and context interchangeably. This is in line with the more general definition of source in the source-monitoring framework (Johnson et al., 1993) that comprises both context features (e.g., spatial position) and internal features (e.g., cognitive operations; Mitchell & Johnson, 2009).

2 The sequential hypothesis states that memories are consolidated in two consecutive steps during slow-wave sleep (SWS) and rapid eye movement (REM) sleep (Ambrosini & Giuditta, 2001; Giuditta, 2014; Giuditta et al., 1995).

3 According to the synaptic homeostasis hypothesis, sleep renormalizes synaptic connections that were strengthened during wakefulness, thereby restoring cellular homeostasis (Cirelli & Tononi, 2015; Tononi & Cirelli, 2003, 2006, 2014, 2020).

4 To check whether our results are confounded by the type of compensation, we conducted additional exploratory analyses based on the analyzed data of Experiment 1 and Experiment 2. Results are provided in the Online Supplemental Materials (OSM; see Tables S2 and S5). Except for a significant interaction of compensation type and wake versus sleep for the false-alarm rate in Experiment 1, there was no statistically significant evidence that item memory (i.e., hit rates, *d’*) and ACSIM-based source memory performance in the wake and sleep conditions were affected by the type of compensation.

5 In both experiments, we calculated the sensitivity index *d’* by *Z*_hit rate_—*Z*_false-alarm rate_ and response bias *c* by -0.5 * (*Z*_hit rate_ + *Z*_false-alarm rate_), with *Z* denoting the probit transformation (see, e.g., Stanislaw & Todorov, 1999). Note that we used the log-linear rule (i.e., adding 0.50 to hits, false alarms, misses, and correct rejections; Hautus, 1995) to obtain estimates of *d’* and *c* even in case of extreme hit rates and false alarm rates of 0 or 1.

6 The result patterns for item and source memory performance did not change when excluding all seven participants from the sleep condition who slept less than 6 h during the retention interval (see Table S3 in the OSM).

7 One participant met the preregistered exclusion criterion “color blindness.” However, the participant reported being red-green color blind. This does not affect discrimination of blue and yellow. Therefore, the participant was not excluded from the analysis.

8 The result patterns for item and source memory performance did not change when excluding all ten participants from the sleep condition who slept less than 6 h during the retention interval (see Table S6 in the OSM).

9 For this reason, we refrain from reporting the preregistered 2HTSM-analyses for separate source dimensions. Not surprisingly given the very low *d* parameters, the separate 2HTSM analyses for spatial position and frame color were highly similar to those for parameters *e*^Position^ and *e*^Color^ in the multidimensional source-monitoring model. For detailed information on the originally planned analyses with the 2HTSM, see the preregistration document on the OSF (https://osf.io/a6z4u).

10 Applying Submodel 5d resulted in the same good fit as in case of Submodel 5a, *G*^2^(2) = 1.78, *p* = .411. For Submodel 5d, we found a statistically significant sleep benefit in item memory (parameter *D*; sleep: MLE = .65, *SE* = .01; wake: MLE = .60, *SE* = .01), Δ*G*^2^(1) = 13.66, *p* < .001. However, whereas a significant sleep benefit emerged in source memory for source “right” (parameter *d*_right_; sleep: MLE = .73, *SE* = .03; wake: MLE = .51, *SE* = .06), Δ*G*^2^(1) = 12.28, *p* < .001, we observed no significant sleep benefit in source memory for source “left” (parameter *d*_left_; sleep: MLE = .46, *SE* = .08; wake: MLE = .45, *SE* = .06), Δ*G*^2^(1) = 0.01, *p* = .919. Concerning the guessing parameters, we found significantly more “old”-guessing in the wake than in the sleep condition (parameter *b*; sleep: MLE = .20, *SE* = .01; wake: MLE = .25, *SE* = .01), Δ*G*^2^(1) = 6.09, *p* = .014, resembling the result observed with Submodel 5a. However, with respect to a stronger “left” guessing bias after sleep (parameter *a*; sleep: MLE = .64, *SE* = .04; wake: MLE = .47, *SE* = .04), Δ*G*^2^(1) = 10.13, *p* = .001, the result differed from the one achieved with Submodel 5a.
