# Supplementary material for: Does sleep benefit source memory? Investigating 12-h retention intervals with a multinomial modeling approach
Source: Mem Cognit. 2024 Jun 3;53(2):467–93. doi: 10.3758/s13421-024-01579-8 (PMC11868154; doi:10.3758/s13421-024-01579-8)
Supplement: Supplementary file 1 — Supplementary file1 (DOCX 52 KB) [file 13421_2024_1579_MOESM1_ESM.docx]

**Supplemental Materials**

accompanying the manuscript *Does Sleep Benefit Source Memory? Investigating 12-hr Retention Intervals with a Multinomial Modeling Approach*

Experiment 1

Table S1 2

Table S2 4

Table S3 5

Experiment 2

Table S4 6

Table S5 8

Table S6 9

References 10

**Table S1**

*Sample Characteristics for Experiment 1*

| Variable | Total | |  | Wake | |  | Sleep | |  |  |
| --- | --- | --- | --- | --- | --- | --- | --- | --- | --- | --- |
|  | *M* | *SD* |  | *M* | *SD* |  | *M* | *SD* | *t*(130) | *p* |
| Age, in years | 26.77 | 4.48 |  | 27.11 | 4.38 |  | 26.45 | 4.58 | 0.85 | .399 |
| OT, buffer plus target | 0.98 | 0.03 |  | 0.97 | 0.03 |  | 0.98 | 0.02 | 2.13 | .035 |
| OT, target only | 0.98 | 0.03 |  | 0.98 | 0.03 |  | 0.99 | 0.02 | 2.30 | .023 |
| SSS, learning session | 2.48 | 0.96 |  | 2.55 | 0.85 |  | 2.40 | 1.06 | 0.90 | .369 |
| SSS, testing session | 2.55 | 1.13 |  | 2.62 | 0.84 |  | 2.48 | 1.35 | 0.70 | .485 |
| rMEQ, sum score | 14.00 | 3.83 |  | 14.15 | 3.79 |  | 13.85 | 3.89 | 0.45 | .651 |
| Retention interval | 11.99 | 0.14 |  | 11.97 | 0.13 |  | 12.01 | 0.15 | 1.44 | .151 |
| Time between learning and sleeping |  |  |  |  |  |  | 3.51 | 1.52 |  |  |
| Sleep duration |  |  |  |  |  |  | 7.37 | 1.19 |  |  |
| Sleep quality |  |  |  |  |  |  | 2.16 | 0.91 |  |  |

*Note*. OT = Orienting task; SSS = Stanford Sleepiness Scale (Hoddes et al., 1973); rMEQ = German version of the reduced Morningness-Eveningness Questionnaire (Randler, 2013). The orienting task records the proportion of correct responses to spatial positions for the items (i.e., 4 buffer and 60 target items). The SSS captures sleepiness on a 7-point scale with higher values indicating greater sleepiness. The rMEQ assesses chronotype with sum scores that are based on five items (Cronbach’s α = .74). Sum scores can range from 4 to 25 with higher values indicating stronger morning preferences. The retention interval, the time between the start of the learning session and the self-reported sleep onset for the sleep condition (*Mdn* = 3.50 hr, range: 0.64–8.63) as well as the self-reported sleep duration (*Mdn* = 7.50 hr, range: 3.00–9.33) in the experimental night are shown in hours. Sleep quality for the experimental night was assessed via self-report on a 5-point scale with higher values indicating better sleep quality. The wake condition contains 65 participants (41 female) of which 40 participants were recruited via Prolific and received payment whereas the sleep condition contains 67 participants (43 female) of which 46 participants were recruited via Prolific. The number of payed participants in the wake and sleep condition does not differ significantly, *t*(130) = 0.85, *p* = .395. In total, two participants (*n*_wake_ = 1, *n*_sleep_ = 1) hold an intermediate school certificate and 130 participants (*n*_wake_ = 64, *n*_sleep_ = 66) hold a qualification for college/university entrance. Whereas 78 participants are college/university students (*n*_wake_ = 38, *n*_sleep_ = 40), 54 participants (*n*_wake_ = 27, *n*_sleep_ = 27) pursue an occupation.

**Table S2**

*Means and Standard Errors for the Memory Performance Measures in Experiment 1 by Type of Compensation*

| Dependent variable | No payment | |  | Payment | |  |  | |  |
| --- | --- | --- | --- | --- | --- | --- | --- | --- | --- |
|  | Wake | Sleep |  | Wake | Sleep | *F*(1,128) | | *p* | η_p_^2^ |
| Hit rate | 0.72 (0.02) | 0.71 (0.02) |  | 0.69 (0.01) | 0.73 (0.01) | 0.47 | .496 | | .00 |
| False-alarm rate | 0.05 (0.01) | 0.07 (0.01) |  | 0.12 (0.13) | 0.07 (0.01) | 4.63 | .033 | | .04 |
| Sensitivity index *d’* | 2.37 (0.08) | 2.21 (0.08) |  | 1.84 (0.09) | 2.29 (0.08) | 3.18 | .077 | | .02 |
| Response bias *c* | 0.49 (0.04) | 0.44 (0.04) |  | 0.36 (0.03) | 0.43 (0.03) | 0.66 | .419 | | .01 |
| ACSIM_Position_ | 0.72 (0.01) | 0.75 (0.01) |  | 0.67 (0.01) | 0.78 (0.01) | 2.32 | .130 | | .02 |

*Note*. Standard errors of the mean are presented in parentheses. Participants were either recruited via Prolifc (86; *n*_wake_ = 40, *n*_sleep_ = 46) or other channels (46; *n*_wake_ = 25, *n*_sleep_ = 21) and were randomly assigned to a wake versus sleep condition. Only participants recruited via Prolific were paid. ACSIM = average conditional source identification measure; *F* = *F* value for the interaction of compensation type and wake versus sleep.

**Table S3**

*Means and Standard Errors for the Memory Performance Measures in Experiment 1 After Exclusion of Participants With Less Than 6 hr of Sleep*

| Dependent variable | Wake | |  | Sleep | |  |  |  |  |
| --- | --- | --- | --- | --- | --- | --- | --- | --- | --- |
|  | *M* | *SE* |  | *M* | *SE* |  | *t*(123) | *p* | Cohen’s **d** [95% CI] |
| Item memory | | | | | | | | |  |
| Hit rate | 0.70 | 0.02 |  | 0.73 | 0.02 |  | 0.94 | .351 | 0.16 [-0.19, 0.51] |
| False-alarm rate | 0.10 | 0.01 |  | 0.07 | 0.01 |  | 1.62 | .109 | -0.29 [-0.64, 0.06] |
| Sensitivity index *d’* | 2.05 | 0.09 |  | 2.31 | 0.08 |  | 1.56 | .121 | 0.28 [-0.08, 0.63] |
| Response bias *c* | 0.41 | 0.03 |  | 0.41 | 0.04 |  | 0.05 | .960 | 0.00 [-0.35, 0.35] |
| Source memory | | | | | | | | |  |
| ACSIM | 0.69 | 0.01 |  | 0.77 | 0.01 |  | 3.40 | .001 | 0.59 [0.23, 0.94] |

*Note*. Means and standard errors of the mean are shown for the wake (*n* = 65) and sleep condition (*n* = 60), as well as the results of two-tailed *t* tests comparing the two independent groups. We calculated Cohen’s **d** on the basis of the means and pooled standard deviations. Note that for both item and source memory, positive values of Cohen’s **d** indicate a sleep benefit, whereas negative values indicate a sleep disadvantage compared to wakefulness. ACSIM = average conditional source identification measure.

**Table S4**

*Sample Characteristics for Experiment 2*

| Variable | Total | |  | Wake | |  | Sleep | |  |  |
| --- | --- | --- | --- | --- | --- | --- | --- | --- | --- | --- |
|  | *M* | *SD* |  | *M* | *SD* |  | *M* | *SD* | *t*(132) | *p* |
| Age, in years | 25.58 | 4.51 |  | 25.11 | 4.49 |  | 25.99 | 4.52 | 1.12 | .265 |
| OT, buffer plus target | 0.97 | 0.06 |  | 0.96 | 0.08 |  | 0.98 | 0.03 | 2.32 | .022 |
| OT, target only | 0.98 | 0.06 |  | 0.96 | 0.07 |  | 0.99 | 0.03 | 2.21 | .029 |
| SSS, learning session | 2.74 | 1.27 |  | 2.68 | 1.13 |  | 2.79 | 1.38 | 0.52 | .605 |
| SSS, testing session | 2.99 | 1.37 |  | 2.98 | 1.26 |  | 3.00 | 1.47 | 0.07 | .946 |
| rMEQ, sum score | 13.08 | 4.04 |  | 12.95 | 3.62 |  | 13.19 | 4.39 | 0.35 | .726 |
| Retention interval | 12.01 | 0.20 |  | 11.98 | 0.22 |  | 12.03 | 0.17 | 1.64 | .104 |
| Time between learning and sleeping |  |  |  |  |  |  | 4.04 | 1.57 |  |  |
| Sleep duration |  |  |  |  |  |  | 6.99 | 1.48 |  |  |
| Sleep quality |  |  |  |  |  |  | 2.26 | 0.89 |  |  |

*Note*. OT = Orienting task; SSS = Stanford Sleepiness Scale (Hoddes et al., 1973); rMEQ = German version of the reduced Morningness-Eveningness Questionnaire (Randler, 2013). The orienting task records the proportion of correct responses to position-color combinations for the items (i.e., 4 buffer and 120 target items). The SSS captures sleepiness on a 7-point scale with higher values indicating greater sleepiness. The rMEQ assesses chronotype with sum scores that are based on five items (Cronbach’s α = .77). Sum scores can range from 4 to 25 with higher values indicating stronger morning preferences. The retention interval, the time between the start of the learning session and the self-reported sleep onset for the sleep condition (*Mdn* = 3.58 hr, range: 1.49–9.44) as well as the self-reported sleep duration (*Mdn* = 7.25 hr, range: 0.50–8.92) in the experimental night are shown in hours. Sleep quality for the experimental night was assessed via self-report on a 5-point scale with higher values indicating better sleep quality. The wake condition contains 62 participants (36 female) of which 43 participants were recruited via Prolific and received payment whereas the sleep condition contains 72 participants (48 female) of which 43 participants were recruited via Prolific. The number of payed participants in the wake and sleep condition does not differ significantly, *t*(132) = 1.16, *p* = .250. In total, the sample includes one pupil (sleep condition), two participants (*n*_wake_ = 1, *n*_sleep_ = 1) with no secondary school certificate, six participants (*n*_wake_ = 3, *n*_sleep_ = 3) with an intermediate school certificate and 125 participants (*n*_wake_ = 58, *n*_sleep_ = 67) with a qualification for college/university entrance. Whereas 70 participants are college/university students (*n*_wake_ = 33, *n*_sleep_ = 37), 64 participants (*n*_wake_ = 29, *n*_sleep_ = 35) pursue an occupation.

**Table S5**

*Means and Standard Errors for the Memory Performance Measures in Experiment 2 by Type of Compensation*

| Dependent variable | No payment | |  | Payment | |  |  | |  |
| --- | --- | --- | --- | --- | --- | --- | --- | --- | --- |
|  | Wake | Sleep |  | Wake | Sleep | *F*(1,130) | | *p* | η_p_^2^ |
| Hit rate | 0.37 (0.02) | 0.44 (0.02) |  | 0.51 (0.02) | 0.58 (0.02) | 0.00 | .944 | | .00 |
| False-alarm rate | 0.13 (0.01) | 0.09 (0.01) |  | 0.14 (0.01) | 0.15 (0.01) | 1.42 | .236 | | .01 |
| Sensitivity index *d’* | 0.87 (0.07) | 1.29 (0.07) |  | 1.28 (0.07) | 1.34 (0.06) | 1.62 | .205 | | .01 |
| Response bias *c* | 0.82 (0.04) | 0.83 (0.05) |  | 0.59 (0.03) | 0.45 (0.03) | 0.84 | .362 | | .01 |
| ACSIM_Position_ | 0.59 (0.01) | 0.70 (0.01) |  | 0.61 (0.01) | 0.64 (0.01) | 2.66 | .105 | | .02 |
| ACSIM_Color_ | 0.58 (0.01) | 0.61 (0.01) |  | 0.53 (0.01) | 0.53 (0.01) | 0.30 | .585 | | .00 |

*Note*. Standard errors of the mean are presented in parentheses. Participants were either recruited via Prolifc (86; *n*_wake_ = 43, *n*_sleep_ = 43) or other channels (48; *n*_wake_ = 19, *n*_sleep_ = 29) and were randomly assigned to a wake versus sleep condition. Only participants recruited via Prolific were paid. ACSIM = average conditional source identification measure; *F* = *F* value for the interaction of compensation type and wake versus sleep**.**

**Table S6**

*Means and Standard Errors for the Memory Performance Measures in Experiment 2 After Exclusion of Participants With Less Than 6 hr of Sleep*

| Dependent variable | Wake | |  | Sleep | |  |  |  |  |
| --- | --- | --- | --- | --- | --- | --- | --- | --- | --- |
|  | *M* | *SE* |  | *M* | *SE* |  | *t*(122) | *p* | Cohen’s **d** [95% CI] |
| Item memory | | | | | | | | |  |
| Hit rate | 0.47 | 0.02 |  | 0.54 | 0.02 |  | 1.87 | .064 | 0.32 [-0.04, 0.67] |
| False-alarm rate | 0.14 | 0.01 |  | 0.13 | 0.01 |  | 0.14 | .892 | -0.08 [-0.44, 0.27] |
| Sensitivity index *d’* | 1.16 | 0.07 |  | 1.34 | 0.07 |  | 1.40 | .165 | 0.24 [-0.11, 0.59] |
| Response bias *c* | 0.66 | 0.04 |  | 0.54 | 0.04 |  | 1.40 | .163 | -0.27 [-0.62, 0.09] |
| Source memory | | | | | | | | |  |
| ACSIM, spatial position | 0.60 | 0.01 |  | 0.66 | 0.01 |  | 2.49 | .014 | 0.48 [0.12, 0.84] |
| ACSIM, frame color | 0.54 | 0.01 |  | 0.55 | 0.01 |  | 0.64 | .523 | 0.10 [-0.26, 0.45] |

*Note*. Means and standard errors of the mean are shown for the wake (*n* = 62) and sleep condition (*n* = 62), as well as the results of two-tailed *t* tests comparing the two independent groups. We calculated Cohen’s **d** on the basis of the means and pooled standard deviations. Note that for both item and source memory, positive values of Cohen’s **d** indicate a sleep benefit, whereas negative values indicate a sleep disadvantage compared to wakefulness. ACSIM = average conditional source identification measure.

**References**

Hoddes, E., Zarcone, V., Smythe, H., Phillips, R., & Dement, W. C. (1973). Quantification of sleepiness: A new approach. *Psychophysiology, 10*(4), 431–436. https://doi.org/10.1111/j.1469-8986.1973.tb00801.x

Randler, C. (2013). German version of the reduced Morningness-Eveningness Questionnaire (rMEQ). *Biological Rhythm Research, 44*(5), 730–736. <https://doi.org/10.1080/09291016.2012.739930>
